# Enhanced carbon loss in the Southern Ocean under mitigation scenarios

**DOI:** 10.1126/sciadv.aee9228

**Published:** 2026-09-02

**Authors:** Huiji Lee, Jong-Seong Kug, Yechul Shin, Dong-Geon Lee, Nadine Goris, Friederike Fröb, Jerry F. Tjiputra, Timothée Bourgeois, Jörg Schwinger

**Affiliations:** ^1^ School of Earth and Environmental Sciences, Seoul National University, Seoul, South Korea.; ^2^ Institute for Sustainable Development, Seoul National University, Seoul, Republic of Korea.; ^3^ Marine Biogeochemistry Division, GEOMAR Helmholtz Centre for Ocean Research, Kiel, Germany.; ^4^ NORCE Research AS, Bjerknes Centre for Climate Research, Bergen, Norway.; ^5^ Geophysical Institute, University of Bergen and Bjerknes Centre for Climate Research, Bergen, Norway.

## Abstract

The Southern Ocean (SO) plays a pivotal role in the global carbon cycle by absorbing anthropogenic carbon dioxide (CO_2_). Yet, it remains uncertain whether this role will persist under future mitigation efforts. Using idealized emission-driven simulations with the Community Earth System Model version 2 (CESM2), we find that the SO transitions from net CO_2_ sink to a substantial source during zero- and negative-emission phases. This outgassing stems from a persistent regional increase in sea surface temperature and a globally uniform decline in surface alkalinity. Together, these changes maintain elevated oceanic partial pressure of CO_2_ (${P\text{co}}_{2}$) despite decreasing atmospheric CO_2_, reversing the air-sea ${P\text{co}}_{2}$ gradient (${dP\text{co}}_{2}$). Moreover, the SO exhibits a high climatological gas-transfer coefficient, primarily driven by the region’s strong wind speeds and high CO_2_ solubility, which amplifies the ${dP\text{co}}_{2}$-driven flux and enhances CO_2_ outgassing. These results indicate that the present-day SO CO_2_ sink may lose its effectiveness during climate mitigation, emphasizing the need to consider regional feedbacks for long-term climate policy effectiveness.

## INTRODUCTION

Since the beginning of industrialization, numerous adverse impacts of global warming have been observed in the climate system, ecosystems, and human societies (*1*–*4*). In response to these climate risks, the 2015 Paris Agreement established an international framework to limit global temperature rise to well below 2°C and to pursue efforts to limit it to 1.5°C above preindustrial levels (*5*). These ambitious goals require not only reaching net-zero emissions but also achieving negative emissions (NEGs), for which carbon dioxide removal (CDR) strategies are essential (*6*–*9*). Assessing the effectiveness and potential risks of CDR pathways necessitates accounting for the complexity of Earth’s climate system (*10*, *11*). Previous studies demonstrate that although many physical climate processes are broadly reversible at global scale, their regional responses can exhibit hysteresis of varying magnitudes (*12*–*16*). In contrast to the relatively comprehensive understanding of physical climate hysteresis, ocean biogeochemical hysteresis remains less well constrained, leaving substantial uncertainty in carbon cycle responses (*17*–*20*). Assessing such hysteresis is critical because the response of ocean systems inherently lags behind mitigation efforts, and these lags can further drive and sustain biogeochemical hysteresis, allowing such changes to persist long after emissions decline and thereby reducing the expected effectiveness of long-term climate mitigation.

The global ocean has absorbed about 30% of anthropogenic carbon dioxide (CO_2_) emissions, buffering the rise of atmospheric CO_2_ concentrations to some extent (*21*–*23*). The Southern Ocean (SO) is a key region for carbon uptake, contributing 40 to 50% of the total mean annual oceanic uptake of anthropogenic CO_2_ (*22*, *24*–*26*). Given its pivotal role in regulating the global climate response, several recent studies have emphasized that improving the understanding of SO carbon cycle dynamics is essential for reducing uncertainties in future climate projection (*27*–*29*). Under global warming scenarios, Earth System Models (ESMs) generally project that most ocean basins will continue to take up CO_2_, although regional responses may differ (*30*–*34*). In particular, the projected strengthening of the SO uptake is driven by warming-induced sea ice reduction, which expands the open-ocean areas available for air-sea CO_2_ gas exchange, and the efficient surface-to-deep transport of carbon through the mode waters (*30*, *35*, *36*).

Although ESMs project an apparently linear response of the SO climate system [e.g., sea surface temperature (SST), mixed-layer depth, and sea-ice cover] to rising CO_2_ (*37*–*39*), assessing its response to atmospheric CO_2_ removal remains a key challenge. Previous studies have shown that the SO physical system responds nonlinearly to CO_2_ removal pathways, with this behavior becoming increasingly pronounced over long-term timescales (*40*–*42*). Such a phenomenon could eventually generate delayed and multifaceted impacts on oceanic carbon dynamics, introducing substantial uncertainty into projections of future CO_2_ flux and the overall impact of climate mitigation. Recent multimodel assessments have demonstrated that the SO exhibits a unique feature characterized by a shift from a CO_2_ sink to source under CO_2_ removal pathways (*43*, *44*). While these broad multimodel trends are becoming established, the underlying mechanisms driving this SO switch and their implications for CO_2_ uptake efficiency under future mitigated conditions remain insufficiently understood. In particular, a critical question is what controls the progressive weakening of the SO carbon sink and the eventual emergence of CO_2_ outgassing under mitigation pathways and what specific processes govern the pace and reversibility of these changes.

In this study, we examine the distinctive carbon cycle response of the SO in the region between 63°S and 47°S in response to CDR strategies. We conduct emission-driven simulations using the state-of-the-art Community Earth System Model version 2 (CESM2) that includes the Marine Biogeochemistry Library (MARBL) model (see Materials and Methods). The simulations follow two idealized emission trajectories: one achieving zero emissions (ZECs) and the other pursuing sustained NEGs (red lines in Fig. 1A; see Materials and Methods). Both simulations are driven by prescribed anthropogenic CO_2_ emissions that increase linearly until 2050 and subsequently decrease while excluding other transient forcings such as aerosols. In the ZEC scenario, emissions stabilize at zero after year 2123, whereas the NEG scenario applies active CO_2_ removal until atmospheric CO_2_ returns to initial levels [∼383 parts per million (ppm)] around 2196 after which emissions remain at zero. Our results reveal that under both mitigation scenarios, the SO shows a pronounced CO_2_ outgassing compared to other regions. While the SO initially serves as a weak CO_2_ sink or remains near neutral under present-day conditions (green lines in Fig. 1B), it transitions into a strong CO_2_ source during the NEG and ZEC phases, indicating a substantial loss of carbon from the ocean reservoir on timescales that are relevant for the design of ambitious mitigation efforts.

**Fig. 1.**
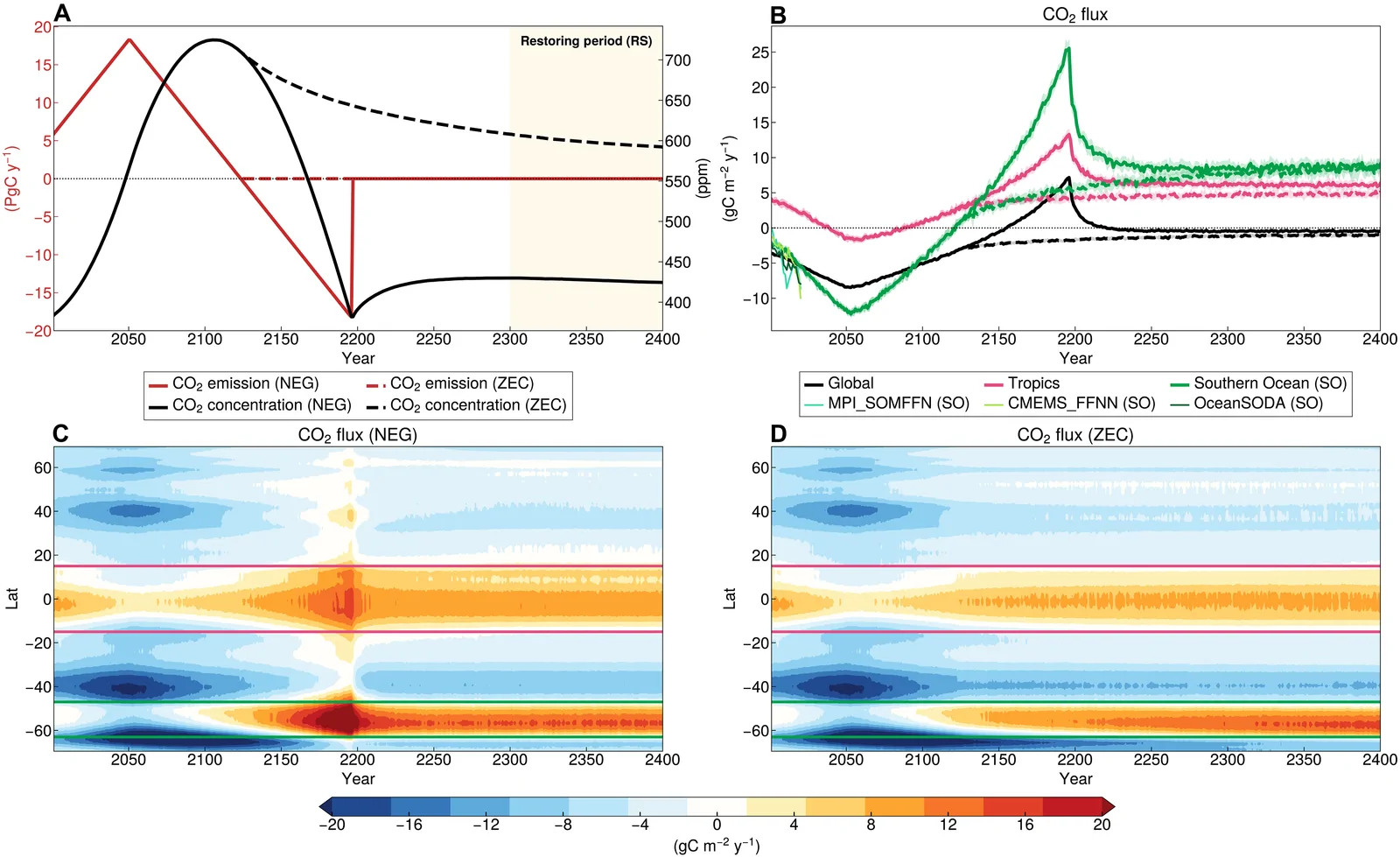
Response of air-sea CO_2_ flux to the climate mitigation scenarios. (**A**) Time series of prescribed annual anthropogenic CO_2_ emissions (red lines) and annual mean atmospheric CO_2_ concentration (black lines). Solid lines represent the NEG experiment, and dashed lines represent the ZEC experiment. y, year. (**B**) Regional time series of the annual mean air-sea CO_2_ flux for global mean (black/gray lines), tropics (pink lines, 15°S to 15°N), and SO (green lines, 63°S to 47°S). Again, the solid lines represent the NEG experiment, and dashed lines represent the ZEC experiment. The lines show the ensemble mean values for each simulation, and the shading indicates the uncertainty of the projections calculated by the interensemble spread (±1 std). The thin green lines indicate the evolution of the SO CO_2_ flux from years 2001 to 2020 on the basis of observed air-sea CO_2_ flux data products, namely MPI-SOMFFN, CMEMS-FFNN, and OceanSODA-ETHZ. (**C** and **D**) Time-latitude diagrams of annual mean air-sea CO_2_ fluxes in the NEG and ZEC experiments, respectively. Negative fluxes represent CO_2_ uptake by the ocean. The pink horizontal lines show the boundaries of the tropics (15°S to 15°N) and the green horizontal lines the boundaries of the SO (63°S to 47°S).

## RESULTS

### Ocean carbon cycle response to mitigation scenarios

Although our idealized simulations are not directly comparable to the recent historical period, the model simulates a globally integrated oceanic CO_2_ uptake over the period of 2001 to 2020, which is similar to present-day observation-based estimates of air-sea CO_2_ fluxes (∼2 PgC year^−1^; fig. S1A) (*22*, *45*, *46*). The temporal evolution of the globally averaged air-sea CO_2_ flux is characterized by enhanced oceanic CO_2_ uptake until the emission peak is reached (approximately year 2050), followed by weakened uptake as the emissions decline (compared to the red lines in Fig. 1A and the black lines in Fig. 1B). In the NEG experiment, this weakening continues, and in the year 2154, the ocean transitions from a net sink to a net source of CO_2_. Following the cessation of NEGs in the year 2196, when atmospheric CO_2_ returns to initial levels, the simulation enters a ZEC phase. During this period, the global mean air-sea CO_2_ flux stabilizes at ∼−0.5 gC m^−2^ year^−1^ (−0.23 PgC year^−1^), indicating continued but diminished CO_2_ absorption when compared to the initial state (solid black line in Fig. 1B and fig. S1B). In the ZEC experiment, the ocean sustains CO_2_ uptake at roughly −0.9 gC m^−2^ year^−1^ (−0.54 PgC year^−1^) from the time emissions cease in 2123 until the end of the simulation (dashed black line in Fig. 1B and fig. S1B). Therefore, in both mitigation scenarios, the global mean air-sea CO_2_ flux remains slightly negative during the restoring period (RS period; years 2300 to 2400), indicating continued but weak oceanic CO_2_ uptake.

CO_2_ flux responses differ substantially across ocean regions (Fig. 1, C and D). At most latitudes, CO_2_ uptake strengthens until the emissions peak and weaken thereafter. During the RS period, air-sea CO_2_ fluxes at these latitudes remain weaker than at the beginning of the simulations. This reflects the continued elevation of atmospheric CO_2_ concentrations relative to the initial level, accompanied by rising oceanic partial pressure of CO_2_ (${P\text{co}}_{2}$), which reduces the air-sea CO_2_ gradient (${dP\text{co}}_{2}$) and therefore constrains further CO_2_ uptake (black lines in Fig. 1A). By contrast, the SO exhibits a distinct response, and the present-day CO_2_ sink turns into a net CO_2_ source around the time emissions approach zero (Fig. 1B and fig. S1D). In detail, this region exhibits a slight CO_2_ uptake at the beginning of the simulation period (∼−2.8 gC m^−2^ year^−1^ and −0.1 PgC year^−1^ during 2001 to 2005), followed by a gradual increase in uptake as emissions increase (green lines in Fig. 1B). However, once CO_2_ emissions are reduced, the uptake declines rapidly, and the SO gradually turns into a net CO_2_ source. During the RS period, the SO emits ∼8.6 gC m^−2^ year^−1^ (0.33 PgC year^−1^) in the ZEC simulation and 8.5 gC m^−2^ year^−1^ (0.35 PgC year^−1^) in the NEG simulation (green lines in Fig. 1B and fig. S1D). Spatially, this reduction in oceanic CO_2_ uptake can also be seen in regions south of 30°S during the RS period, but the SO exhibits the most pronounced response (fig. S2). By contrast, the tropics (15°S to 15°N) release about 4.8 gC m^−2^ year^−1^ (0.6 PgC year^−1^) in ZECs and 6 gC m^−2^ year^−1^ (0.8 PgC year^−1^) in NEGs during this time (pink lines in Fig. 1B and fig. S1C). When area-integrating the CO_2_ flux, the tropics account for a slightly larger total CO_2_ source because of their larger spatial extent (fig. S1C). However, in terms of changes, the area-integrated ∆air-sea CO_2_ flux shows a substantially larger increase in the SO relative to the tropics (fig. S1, F and G). This indicates that the SO undergoes more marked changes and contributes disproportionately to the global outgassing signal under the mitigation experiments. This regional transition affects not only the Southern Hemisphere but also the global carbon budget. Next, we will focus on the mechanisms driving this transition of the SO CO_2_ flux from a sink to source under the NEG and ZEC conditions.

### Contributions to *P*co_2_ changes in the SO

The direction of air-sea CO_2_ fluxes is primarily determined by the difference between the atmospheric and oceanic CO_2_ partial pressures: ${\mathrm{dP}\text{co}}_{2}$ (*47*, *48*). As the atmospheric CO_2_ partial pressure (${P\text{co}}_{2}^{\text{atm}}$) is nearly globally uniform (fig. S3), most spatial variations in ${\mathrm{dP}\text{co}}_{2}$ reflect regional differences in oceanic CO_2_ partial pressure (${P\text{co}}_{2}^{\text{ocn}}$). In our simulations, ${P\text{co}}_{2}^{\text{atm}}$ exceeds ${P\text{co}}_{2}^{\text{ocn}}$ in the SO until the CO_2_ concentration peak is reached (year 2107), facilitating continuous oceanic CO_2_ uptake in this region [compare solid and dashed black lines in Fig. 2 (A and C)]. However, after the peak, ${P\text{co}}_{2}^{\text{ocn}}$ surpasses ${P\text{co}}_{2}^{\text{atm}}$ and remains higher, resulting in a sustained positive ${\mathrm{dP}\text{co}}_{2}$ that translates to the SO having become an ocean CO_2_ source.

**Fig. 2.**
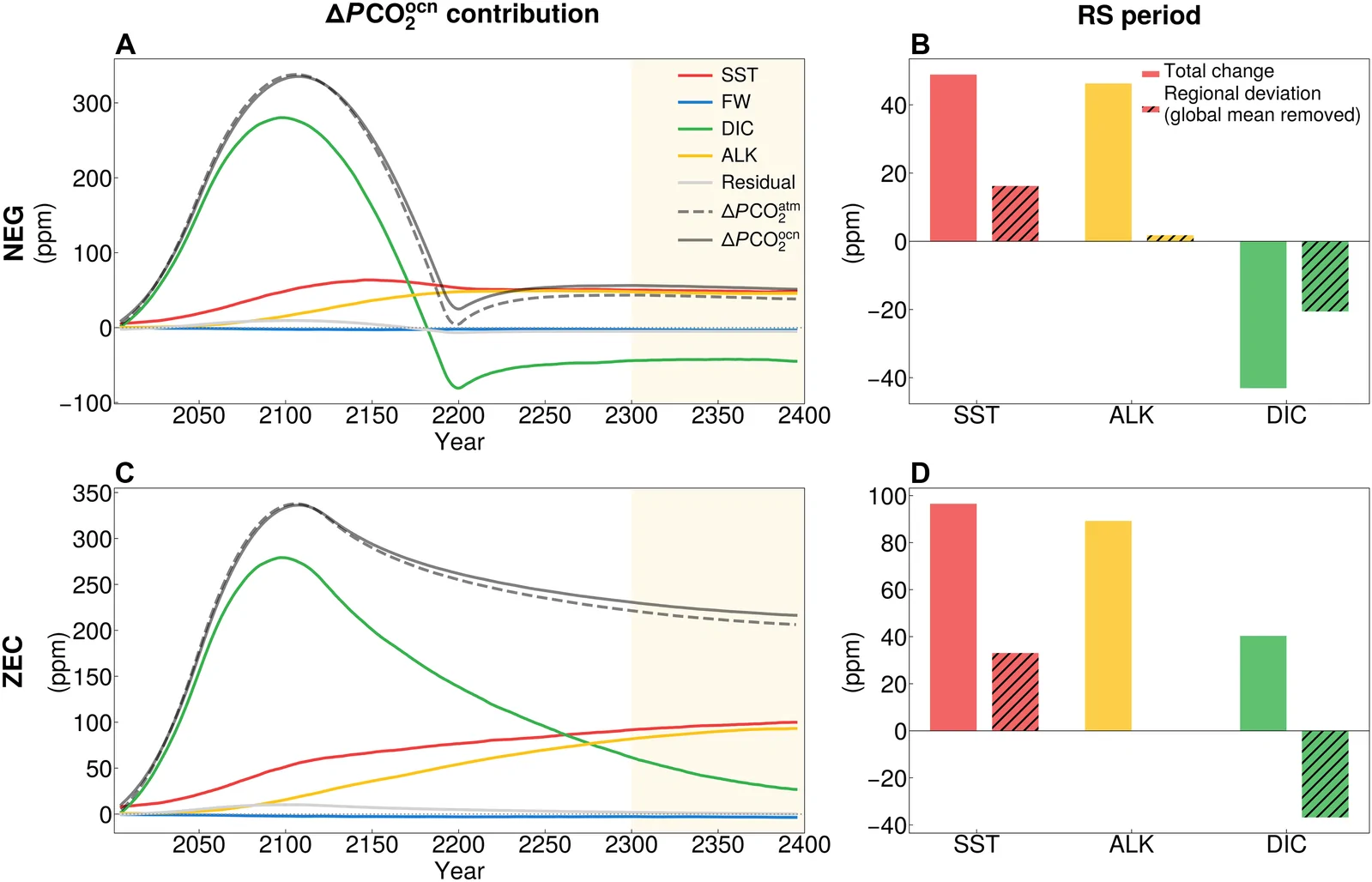
Contributions of ${P\text{co}}_{2}^{\text{ocn}}$ changes in the SO. (**A** and **C**) Temporal evolution of contributions of $∆P{\text{co}}_{2}^{\text{ocn}}$ (the change of $P{\text{co}}_{2}^{\text{ocn}}$ relative to the initial 5-year mean of experiment) in the SO under each experiment. The total $∆P{\text{co}}_{2}^{\text{ocn}}$ and $∆{P\text{co}}_{2}^{\text{atm}}$ are represented by the black solid and dashed lines, respectively. The colored lines indicate the ensemble mean contributions of changes in temperature (SST; red), surface DIC (green), surface total ALK (yellow), and FW (blue) to $∆P{\text{co}}_{2}^{\text{ocn}}$ (shadings indicate the interensemble spread ±1 std). DIC and ALK are averaged over the upper ocean (0 to 100 m), and they are normalized by salinity (the standard salinity is 35 psu). (**B** and **D**) Average over the past 100 years of the scenarios [RS period, which is yellow shading in (A) and (C)] for SST, ALK, and DIC contributions. The hatched bar indicates the values after the global mean is removed (SO contribution minus global mean contribution, which is called the anomaly contribution).

To understand the oceanic processes responsible for this CO_2_ flux evolution in the SO region, we decompose its ${P\text{co}}_{2}^{\text{ocn}}$ changes ($∆{P\text{co}}_{2}^{\text{ocn}}$) into a thermal component, i.e., changes driven by SST, and nonthermal components, i.e., changes driven by dissolved inorganic carbon (DIC), alkalinity (ALK), and freshwater (FW) (see Materials and Methods) (*49*). This method allows us to identify the main factors driving the increase in the SO $∆{P\text{co}}_{2}^{\text{ocn}}$ [the residuals of our decomposition remain reasonably small; see light gray lines in Fig. 2 (A and C)]. Our results show that the FW contributions to $∆{P\text{co}}_{2}^{\text{ocn}}$ are negligible in the SO region in both experiments [blue lines in Fig. 2 (A and C)], but we note that the Antarctic ice sheet is not represented in our model. DIC-driven changes in ${P\text{co}}_{2}^{\text{ocn}}$ closely follow the atmospheric CO_2_ trajectories [green lines in Fig. 2 (A and C)]. During the phases where atmospheric CO_2_ declines, the role of DIC diminishes markedly in the ZEC experiment, and it even acts to reduce $∆{P\text{co}}_{2}^{\text{ocn}}$ in the NEG experiment. Conversely, changes in SST and ALK consistently contribute to an increase in SO $∆{P\text{co}}_{2}^{\text{ocn}}$ throughout the simulations [red and yellow lines in Fig. 2 (A and C)]. For the RS phases of each experiment, SST and ALK show pronounced positive contributions to the $∆{P\text{co}}_{2}^{\text{ocn}}$ of the SO region, whereas the contribution of DIC remains minor and even exerts a negative influence.

To determine which factor contributes most to the distinct SO response, it is essential to evaluate each driver relative to its global mean influence. By removing the global mean contribution of each driver, we compare the importance of each driver in the SO relative to its global influence, thereby isolating the regionally dominant processes that most strongly increase SO $∆{P\text{co}}_{2}^{\text{ocn}}$ [referred to here as anomaly contribution; hatched bars in Fig. 2 (B and D)]. During the RS period, the contribution of SST is markedly larger in the SO region than in the global mean [red hatched bars in Fig. 2 (B and D)]. Even as atmospheric CO_2_ declines, global SST remains elevated with a spatially heterogeneous pattern [Fig. 3 (A and B)]. Notably, enhanced surface warming is evident not only in the tropics but also in the SO. By the end of the simulations, regional deviations of the SO SST changes in the NEG and ZEC experiments are ∼0.25° and 1°C above the global mean, respectively (Fig. 3C). This persistent surface warming reflects the gradual release of heat previously stored in the ocean interior during the global warming phase, occurring over decadal to centennial timescales as atmospheric CO_2_ levels decline (*41*, *50*). During this process, low-cloud feedback (*51*, *52*) and sea-ice-albedo feedback (*53*–*55*) play important roles in impeding the efficient release of ocean heat into the atmosphere, thereby amplifying surface warming. In particular, the reduction in low-level cloud cover increases incoming shortwave radiation, while the loss of sea ice lowers the surface albedo, further enhancing solar heat absorption. Therefore, the sustained and intensified ocean surface warming reduces CO_2_ solubility and alters the carbonate equilibrium, thereby enhancing oceanic CO_2_ outgassing in the SO.

**Fig. 3.**
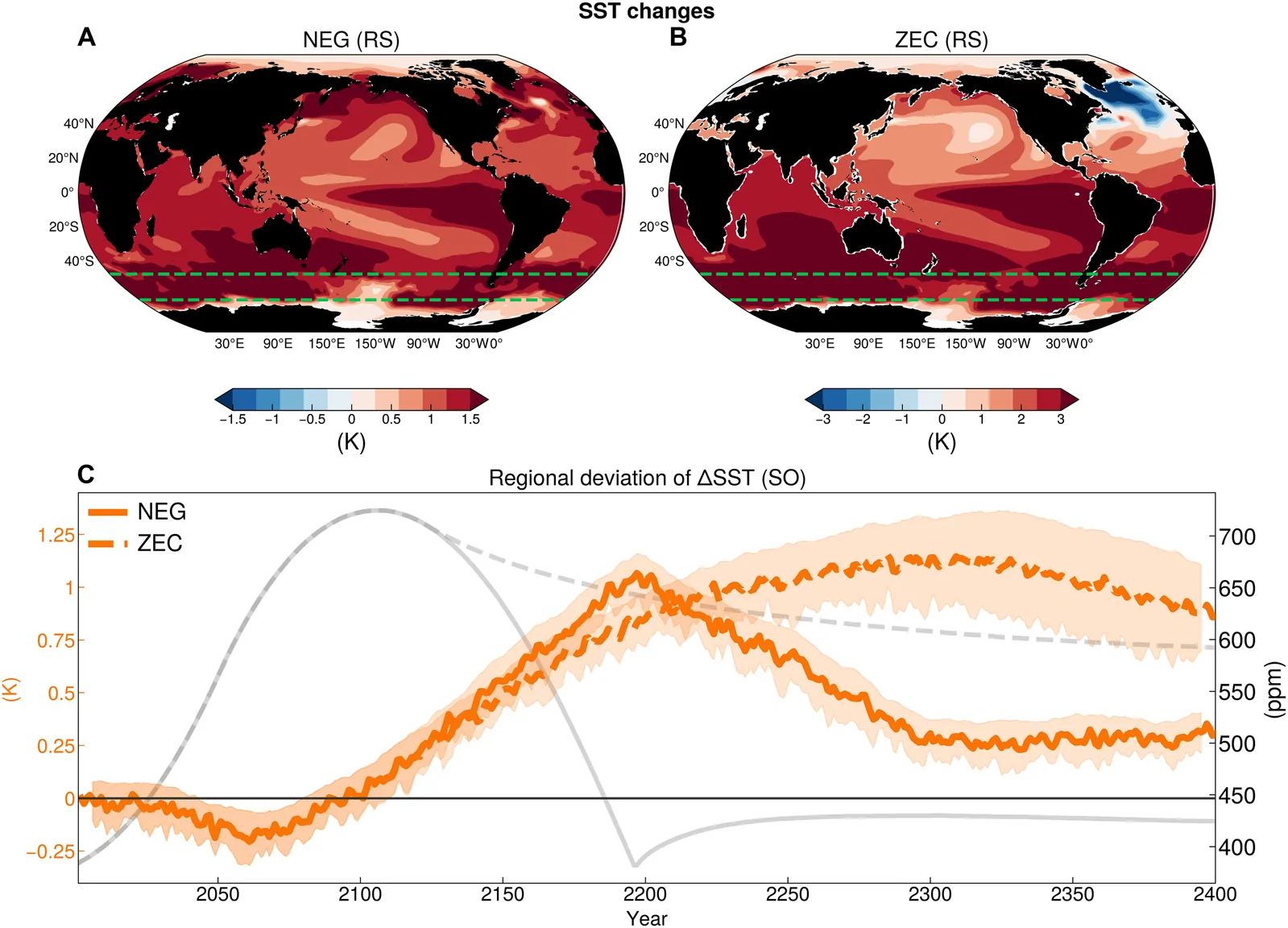
SO SST response under CO_2_ mitigation. (**A** and **B**) SST changes in the past 100-year average of each experiment (RS period), relative to the mean over the initial 5 years. The green dashed lines indicate the SO boundaries. (**C**) Temporal evolution of SST anomaly in the SO relative to the global mean in the NEG (solid line) and ZEC (dashed line) experiments. Lines represent the ensemble mean, and shading shows the interensemble spread (±1 std). A zero SO anomaly of SST changes (black line) indicates that the SO experiences the same SST change as the global ocean. Gray lines (right *y* axis) indicate the atmospheric CO_2_ concentration in NEGs (solid line) and ZECs (dashed line), respectively.

ALK acts as a secondary contributor to the increase in SO $∆{P\text{co}}_{2}^{\text{ocn}}$ through a decline in the surface salinity-normalized ALK (sALK) across large parts of the global ocean [yellow lines in Fig. 2 (A and C); fig. S4]. However, because these ALK changes are widespread rather than specific to the SO, the regional ALK anomaly contribution is negligible during the RS period, indicating that ALK contributes little to distinguishing the SO response from the global mean [yellow hatched bars in Fig. 2 (B and D)]. The widespread ALK reduction is primarily driven by sustained biological exports and limited physical resupply from the deep ocean. Throughout the SO south of 30°S, net primary production, particularly by small phytoplankton, continues to increase under favorable conditions (*56*, *57*), promoting elevated CaCO_3_ production (fig. S5). This intensified biological pump sequesters ALK into the poorly ventilated deep ocean—a phenomenon known as “ALK trapping” (fig. S6) (*58*, *59*). Simultaneously, climate-induced increases in upper-ocean stratification further reduce the vertical transport and upwelling of this ALK-rich deep water back to the surface. Consequently, declining surface ALK weakens the carbonate buffering capacity and reduces the efficiency of oceanic CO_2_ uptake (*60*, *61*). However, because this reduction occurs over large parts of the global ocean, it contributes little to the SO anomaly relative to the dominant SST effect.

The anomalously low SO surface DIC concentration compared to the global mean further corroborates the dominant role of SST-driven outgassing. The intensified SO warming described above drives enhanced CO_2_ outgassing, which in turn reduces surface DIC through thermodynamic carbon loss. The reduction in surface ALK additionally suppresses carbonate buffering, further facilitating CO_2_ outgassing and the associated loss of surface DIC. This physical removal of carbon, reflected in a DIC anomaly contribution of similar magnitude to the positive SST effect [green hatched bars in Fig. 2 (B and D)], implies that the upwelling of waters enriched with anthropogenic DIC is not the primary driver of SO CO_2_ outgassing relative to the global mean. Consequently, our findings suggest that regionally intensified sea surface warming plays a dominant role in the enhanced CO_2_ outgassing in this region, with the globally widespread decline in ALK acting as a secondary contributor.

### Emergence of gas transfer sensitivity as a key modulator of CO_2_ uptake

Our findings demonstrate that persistent regional warming in the SO plays a crucial role in increasing the $∆{P\text{co}}_{2}^{\text{ocn}}$ and constitutes a key mechanism for promoting CO_2_ outgassing. However, comparable thermal conditions are found in other regions, particularly within the tropics (15°S to 15°N), where SST remains ∼1.2°C (NEG) and 2.7°C (ZEC) during the RS period, respectively (Fig. 3, A and B). Hence, the pronounced CO_2_ outgassing in the SO cannot be explained by SST warming alone and must involve additional processes. Zonal-mean ${\mathrm{dP}\text{co}}_{2}$ and CO_2_ flux during the RS period reveal that most regions (e.g., 45°S to 20°S and north of 30°N) remain net CO_2_ sinks with a negative ${\mathrm{dP}\text{co}}_{2}$ (Figs. 1, C and D, and 4A). In contrast, positive ${\mathrm{dP}\text{co}}_{2}$ emerges over the major warming zones—the tropics and the SO. While the tropics show the largest positive ${\mathrm{dP}\text{co}}_{2}$, the associated CO_2_ outgassing is relatively weak. Conversely, the SO shows a more moderate positive ${\mathrm{dP}\text{co}}_{2}$ but a substantial net CO_2_ release to the atmosphere. This contrast highlights that, although ${\mathrm{dP}\text{co}}_{2}$ is the primary driver of the sign of the air-sea CO_2_ flux, it alone cannot fully account for the strength of the flux.

**Fig. 4.**
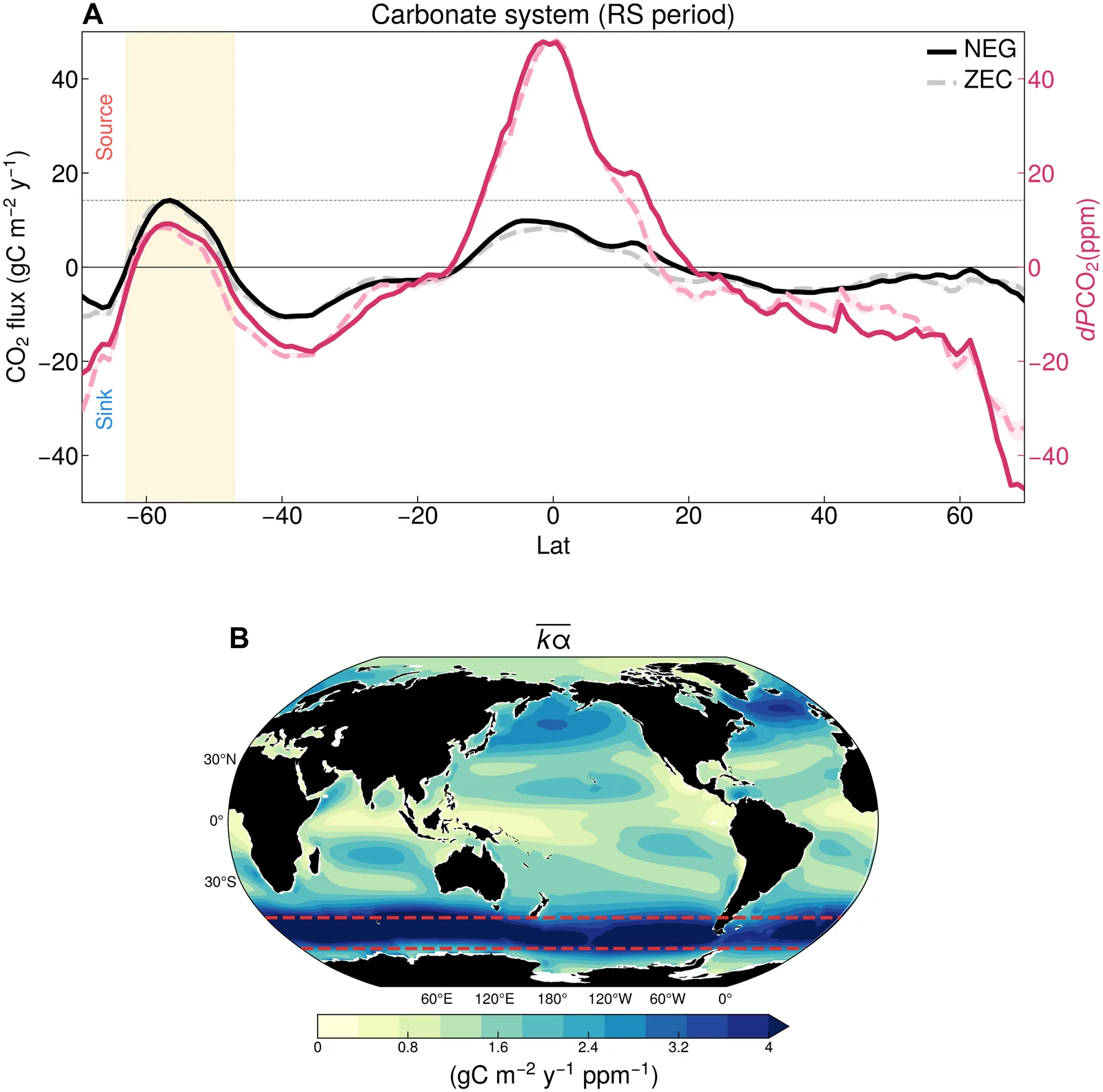
SO CO_2_ outgassing linked to high gas transfer coefficient during mitigation. (**A**) Latitudinal CO_2_ flux (black) and ${\mathrm{dP}\text{co}}_{2}$ (pink) values averaged over the past 100 years of each experiment (RS period). Solid lines represent the NEG experiment, and dashed lines represent the ZEC experiment. Lines represent the ensemble mean, and shading shows the interensemble spread (±1 std). Note that the shading may be difficult to distinguish visually because of the very small magnitude of the spread. Yellow shading indicates the range of the SO. (**B**) Mean gas transfer coefficient climatology ($\bar{k\alpha }$), averaged for the initial 5 years of the experiment. The dashed red lines indicate the SO boundaries.

Given that the air-sea CO_2_ flux depends not only on ${\mathrm{dP}\text{co}}_{2}$ but also on environmental factors such as wind speed and solubility (*62*, *63*), we further calculate the gas transfer coefficient (kα), defined as the product of gas transfer velocity (k) and CO_2_ solubility (α), and apply a linear decomposition of CO_2_ flux changes (see Materials and Methods). Note that, although wind speed strongly influences the gas transfer coefficient, the influence of climate-driven changes in wind speed itself is negligible in our simulations (green line in the fig. S7). This decomposition highlights the importance of the gas transfer coefficient kα in amplifying the flux response. The SO, climatologically characterized by strong westerly winds and high solubility, exhibits $\bar{k\alpha }$ values during 2001 to 2005 nearly twice as large as the global mean (∼4 gC m^−2^ year^−1^ ppm^−1^ compared to the global mean of ∼2 gC m^−2^ year^−1^ ppm^−1^) in our experiments, thereby effectively doubling the flux sensitivity to ${\mathrm{dP}\text{co}}_{2}$.

Once atmospheric CO_2_ begins to decline and stabilize in our experiments, the delayed adjustments of SST and ALK elevate ${P\text{co}}_{2}^{\text{ocn}}$ above ${P\text{co}}_{2}^{\text{atm}}$, yielding a small but positive ${\mathrm{dP}\text{co}}_{2}$ during the ZEC period. Although this increase is modest, the role of $\bar{k\alpha }$ becomes decisive. As shown in Fig. 4B, the SO has the highest $\bar{k\alpha }$ values among all basins, thereby amplifying the positive ${\mathrm{dP}\text{co}}_{2}$-driven flux and resulting in notable CO_2_ outgassing. The spatial distribution of $\bar{k\alpha }$ shows elevated values in regions of low SST (high solubility) and strong winds, with the SO standing out as the region with the highest values (dashed red line in Fig. 4B). By contrast, the tropics—characterized by weaker winds and lower solubility—exhibit lower $\bar{k\alpha }$, resulting in only modest CO_2_ outgassing despite the largest positive ${\mathrm{dP}\text{co}}_{2}$. Consequently, the SO undergoes a distinct and intensifying positive flux anomaly (fig. S2), whereas CO_2_ uptake in most ocean basins merely diminishes in magnitude as emissions decline. This local CO_2_ outgassing grows in magnitude over time—particularly in the ZEC simulation—despite atmospheric CO_2_ remaining at elevated levels toward the end of the simulation (dashed line in Fig. 1A). This sustained and increasing release of CO_2_ indicates that the SO not only fails to retain its initial sink capacity but evolves into an increasingly dominant source that offsets global mitigation efforts.

### Hysteresis of factors contributing to the SO air-sea CO_2_ flux

Thus far, we have found that both the surface ALK and SST contributions to $∆P{\text{co}}_{2}^{\text{ocn}}$ keep increasing during our simulations, leading to a rise in regional $∆P{\text{co}}_{2}^{\text{ocn}}$ regardless of whether atmospheric CO_2_ is reduced or stabilized. Such behavior implies pronounced hysteresis with respect to CO_2_ forcing. Despite declining atmospheric CO_2_, surface ALK shows a clear hysteresis with the recovery trajectory failing to retrace the pathway of increasing CO_2_ (Fig. 5A), suggesting irreversible changes that persist at least until the year 2400 under the mitigation scenarios. It continues to decrease, reaching surface concentrations that are ∼11 mmol m^−3^ lower in the NEG and 25 mmol m^−3^ lower in the ZEC compared to the initial state, driven by the coupled effects of continuous biological export and reduced physical resupply. Also, the surface ALK changes in the global mean and the SO are comparable, yielding similarly extensive hysteresis loops (black and green lines in Fig. 5A).

**Fig. 5.**
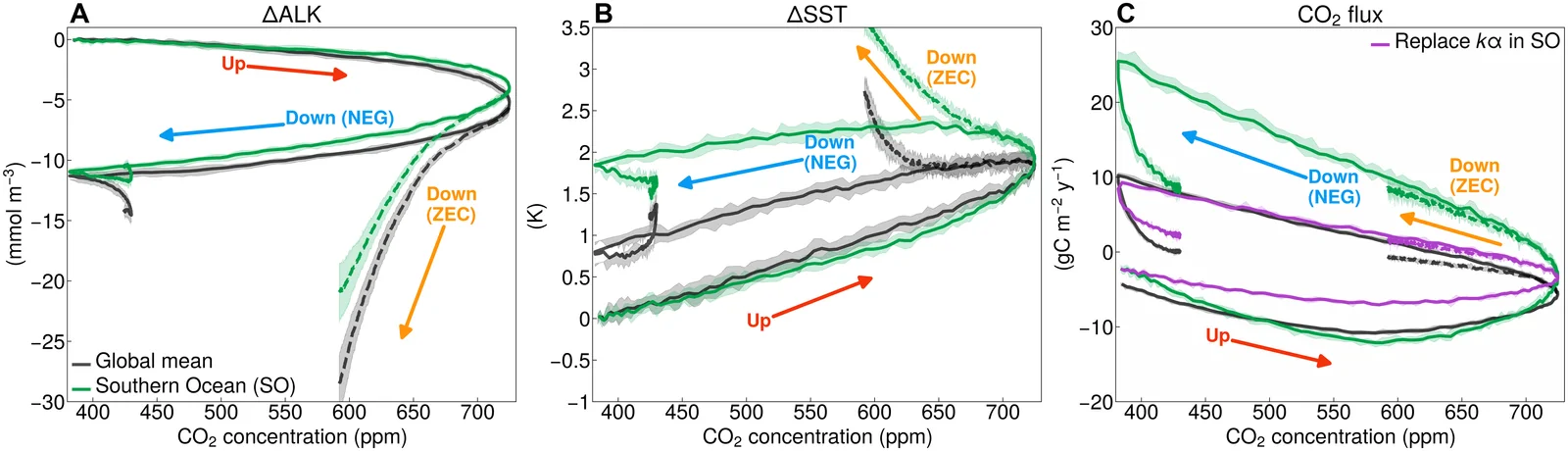
Hysteresis in ALK, SST, and CO_2_ flux responses to atmospheric CO_2_. Annual mean (**A**) ALK changes, (**B**) SST changes relative to the initial 5 years, and (**C**) air-sea CO_2_ flux as a function of CO_2_ concentration for the global mean (black), SO (green), and SO using global mean kα (purple). Note that the kα-replaced CO_2_ flux represents the SO CO_2_ flux recalculated by replacing the regional kα with the global mean. The up period (red arrows) is from 2001 to 2108, and the down period (blue arrows for NEGs and orange arrows for ZECs) is from 2109 to 2400. Solid lines represent the NEG experiment, and dashed lines represent the ZEC experiment. Lines indicate the ensemble mean, and shadings are the interensemble spread (±1 std). ALK is averaged over the upper ocean (0 to 100 m) and normalized by salinity (the standard salinity is 35 psu).

Likewise, SST exhibits a distinct open-loop hysteresis but with regional deviations from the global mean response depending on the type of mitigation (Fig. 5B). During the period when atmospheric CO_2_ is rising, SST increases both globally and in the SO by ∼1.7°C. This leads to the SST of the SO remaining roughly 1.0°C higher than the global mean, as also reflected in the SST pattern during the RS period (Fig. 3, A and B). Therefore, the regionally increased SST response, coupled with the globally uniform decline in surface ALK, sustains elevated regional $∆P{\text{co}}_{2}^{\text{ocn}}$ levels and therefore facilitates the prolonged SO CO_2_ outgassing in our mitigated scenarios.

Both the global and SO CO_2_ fluxes exhibit notable hysteresis with respect to atmospheric CO_2_ concentration (Fig. 5C). During the CO_2_ ramp-up period, the SO uptake strengthens roughly in line with the global uptake. During the CO_2_ ramp-down period, however, the SO trajectories of air-sea CO_2_ fluxes differ from the global pathway such that the SO releases nearly 2.5 times more CO_2_ than the global mean when atmospheric CO_2_ concentrations have returned to their initial levels. The stronger response in the SO compared to the global mean results from enhanced regional SST warming combined with a high gas transfer coefficient, which amplifies the flux response to small positive ${\mathrm{dP}\text{co}}_{2}$.

To better understand the influence of kα, we test how the SO air-sea CO_2_ flux would change if the regional kα is replaced with the global mean (see Materials and Methods for details). This substitution leads to a considerable contraction of the SO hysteresis area (purple line in Fig. 5C). By the end of atmospheric CO_2_ decrease in the NEG experiment, the magnitude of the SO flux closely approaches the global mean (black and purple lines in Fig. 5C). This finding emphasizes that the SO CO_2_ outgassing, initially triggered by a modest positive ${\mathrm{dP}\text{co}}_{2}$, is strongly amplified by the region’s unique kα. Therefore, future assessments of long-term mitigation strategies should consider the regional environmental controls on the gas transfer coefficient (kα), as this factor regulates the magnitude of CO_2_ flux in conjunction with ${\mathrm{dP}\text{co}}_{2}$ and is essential for accurately projecting the future ocean carbon cycle.

## DISCUSSION

This study investigates the SO carbon cycle response under two idealized emission pathways: a pathway that reaches (and maintains) ZECs and a pathway that reduces atmospheric CO_2_ to lower levels through NEGs, also followed by a phase of ZECs. Although the global mean ocean remains a weak net CO_2_ sink in both scenarios during the ZEC phases (black lines in Fig. 1B), the SO shifts from an initial CO_2_ sink (−2.8 gC m^−2^ year^−1^ in year 2001) to a substantial CO_2_ source (+8 to +9 gC m^−2^ year^−1^ by year 2400). Notably, this outgassing exceeds that of the tropics, where substantial CO_2_ release occurs under present-day conditions. On the basis of the decomposition of the $∆P{\text{co}}_{2}^{\text{ocn}}$, we find that increasing regional SST, combined with a global decline in surface ALK, elevates the $∆P{\text{co}}_{2}^{\text{ocn}}$ in the SO. These effects generate a slightly positive ${\mathrm{dP}\text{co}}_{2}$ as atmospheric CO_2_ concentrations decline, thereby initiating CO_2_ outgassing.

However, the positive ${\mathrm{dP}\text{co}}_{2}$ alone cannot explain the large magnitude of the CO_2_ flux, given that the SO exhibits stronger CO_2_ outgassing than the tropics, even though the tropics display the largest positive ${\mathrm{dP}\text{co}}_{2}$ (pink lines in Fig. 4A). During the RS period, the SO’s high gas-transfer coefficient plays a decisive role in amplifying the ${\mathrm{dP}\text{co}}_{2}$-driven flux response. Owing to strong westerly winds and high solubility, the climatological gas transfer coefficient $\bar{k\alpha }$ in the SO is nearly twice the global mean, effectively doubling the air-sea CO_2_ flux. As a consequence, even a small positive ${\mathrm{dP}\text{co}}_{2}$ produces disproportionately large CO_2_ outgassing in the SO.

This CO_2_ loss from the SO during the ZEC and NEG phases exerts a considerable impact on atmospheric CO_2_ concentrations. When the integrated area-based fluxes are expressed in ppm units, the cumulative CO_2_ flux in the SO reaches a net release of ∼30 ppm (+75 PgC) of CO_2_ in the NEG scenario and 8 ppm (+38 PgC) in ZEC scenario (fig. S8). Yet, if we estimate the SO carbon loss by applying the global mean value of kα, we find a strongly reduced cumulative SO CO_2_ flux, corresponding to a release of about 3 ppm (+5 PgC) in the NEG scenario and even a net uptake of roughly 9 ppm (−15 PgC) in the ZEC scenario. These results show that substituting the strong regional kα with a global mean value substantially weakens the SO CO_2_ release, indicating that the high kα in this region continues to amplify outgassing even as atmospheric CO_2_ declines, thereby posing a potential limitation to the long-term efficacy of carbon removal strategies.

Future projections of the SO carbon uptake from ESMs remain highly uncertain, largely due to the challenges in accurately representing complex dynamical and biogeochemical processes in the SO (*27*, *28*). Consequently, the projected oceanic CO_2_ uptake under future climate mitigation scenarios is expected to show intermodel diversity (*48*). While ALK projections are particularly uncertain across CMIP6 models (*64*, *65*), the ALK response in CESM2 is supported by its reasonable representation of the ocean’s chemical state (*66*). Given the impact of surface ALK on CO_2_ fluxes identified in our results (Fig. 2A), it is essential to validate these model-driven findings through observational evidence. We therefore emphasize the necessity for sustained, multidecadal monitoring of the SO carbonate system, including ALK, to reduce current model uncertainties and confirm the enduring effectiveness of climate mitigation strategies. In contrast, SO warming emerges as a robust feature across the ESMs in global warming scenarios, implying that the warming may still manifest under mitigation scenarios, albeit with differing magnitudes among models (*13*, *41*, *67*–*70*). This consistency reinforces confidence in the mechanisms identified in this study as the primary drivers of the positive ${\mathrm{dP}\text{co}}_{2}$ response.

We examine the CO_2_ flux responses in simulations of the Zero Emissions Commitment Model Intercomparison Project (ZECMIP) (*71*). Following the cessation of emissions, the SO exhibits an enhanced CO_2_ outgassing over time (fig. S9). Although there is a considerable model spread, all models show a very similar temporal pattern with increasing CO_2_ outgassing over time. The diversity in the strength of the SO CO_2_ outgassing across ZECMIP models may arise from differences in the intensity of SO warming and from uncertainties in the projected Southern Hemisphere westerlies (*72*), which in turn influence variations in the gas transfer coefficient. Accordingly, additional multimodel assessments are needed to better constrain these mechanisms and to evaluate their roles in determining future SO fluxes under the long-term mitigation scenarios.

Most regions of the SO have historically acted as a critical regulator of the global climate system by absorbing a substantial fraction of anthropogenic CO_2_ emissions (*73*). Many future climate projections indicate that this strong uptake will persist under continued emissions, reinforcing the expectation that this region will remain an important buffer against future warming (*28*, *29*). However, our findings reveal that this behavior may not hold once emissions decline. Under ambitious CO_2_ mitigation, the SO exhibits a progressive and anomalous intensification of CO_2_ outgassing over time, evolving into a substantial carbon source that grows even as atmospheric concentrations fall. This feature highlights that the SO could actively impede global efforts to reach and maintain net-zero targets. Such a unique and persistent feedback underscores the need to reduce uncertainties in the SO carbon cycle processes through improved observation monitoring and model representation to support robust and reliable assessments of climate mitigation strategies.

## MATERIALS AND METHODS

### Experimental design

We used CESM2 to simulate two idealized CO_2_ emission–driven scenarios. CESM2 couples physical model components (atmosphere, ocean, land, and sea ice), with land and ocean carbon cycles (*74*). In particular, the model comprises Community Atmosphere Model version 6 (CAM6), Parallel Ocean Program version 2 (POP2) (*74*, *75*), Community Ice CodE version 5 (CICE5) (*76*) for sea ice, and Community Land Model version 5 (CLM5) (*77*). Ocean biogeochemistry within CESM2 is represented through the MARBL (*66*), encompassing prognostic carbonate chemistry, nutrient cycles, multiple phytoplankton functional groups, and zooplankton dynamics.

Two idealized scenarios have been developed to explore gradual emission reductions and a pathway with CDR. These scenarios differ on the basis of whether emissions transition directly to zero (ZECs) or undergo a temporary negative phase before reaching zero (NEGs) (red lines in Fig. 1A). Anthropogenic CO_2_ emissions in both experiments increase linearly over 2001 to 2050, roughly aligning with the CO_2_ emission rate of the SSP5-8.5 scenario (17 PgC year^−1^ in 2050) (*78*). In our two experiments, emissions decrease subsequently symmetrically at the same rate until 2123. Beyond 2123, the ZEC experiment maintains emissions at zero until the end of the simulation in 2400. In contrast, in the NEG experiment, NEGs are applied from year 2123, implemented as direct air capture continuing the prior linear declining emission rate. NEGs stop at a maximum rate of −18 PgC year^−1^ when atmospheric CO_2_ concentrations reach initial levels (∼383 ppm) around year 2197, after which emissions are held constant at zero until the simulation ends. The atmospheric CO_2_ concentration increases exponentially with rising anthropogenic emissions, peaking in 2107. In the ZEC experiment, CO_2_ concentrations then decline gradually, reaching ∼600 ppm in 2400 (dashed black line in Fig. 1A). In the NEG case, the CO_2_ concentration continues to decline until it returns to the initial level in 2197 when NEGs cease. Thereafter, it stabilizes at around 430 ppm until the end of the simulation (solid black line in Fig. 1A).

These simulations were conducted using an emission-driven model setup, which means that the atmospheric CO_2_ concentration evolves according to prescribed anthropogenic CO_2_ emissions and interactive exchange with the ocean and the land biosphere, thereby including changes in atmospheric CO_2_ concentration resulting from carbon cycle feedbacks. We conducted a total of 10 ensemble members with slightly different initial conditions for each experiment, and all model datasets are regridded to a horizontal resolution of 1° by 1°.

### Air-sea CO_2_ flux data–based products

For the estimation of the contemporary air-sea CO_2_ flux of the open ocean, we examined the observation-based air-sea CO_2_ flux data from the Max Planck Institute for Meteorology Self-Organizing Map Feed Forward Neural Network (MPI-SOMFFN) (*79*), OceanSODA-ETHZ (*80*), and Copernicus Marine Environment Monitoring Service Fast-Four-Neural Network (CMEMS-FFNN) (*81*), used for the years 2001 to 2020.

### Estimates of the air-sea CO_2_ flux

To be able to decompose the modeled CO_2_ fluxes into contributions from different factors, we estimated the air-sea CO_2_ flux ($F_{\text{CO}2}$) using the gas exchange formulation as follows (*82*)

$$F_{\text{CO}2}=k\times \alpha \times {\mathrm{dP}\text{co}}_{2}$$ (1)

where α represents the CO_2_ solubility in seawater, k is the gas exchange velocity, and ${\mathrm{dP}\text{co}}_{2}$ is the difference in CO_2_ partial pressure between the ocean and the atmosphere. Their detailed calculation method of eq. S1 is available in text S1. Note that, in this study, the flux estimation is limited to the global oceanic region between 65°S and 65°N, excluding areas covered by sea ice (fig. S11). Here, negative $F_{\text{CO}2}$ values indicate CO_2_ uptake by the ocean from the atmosphere, whereas positive values indicate CO_2_ release from the ocean to the atmosphere.

To assess the accuracy of the reconstruction, we compared the results with $F_{\text{CO}2}$ directly calculated from the model. The $F_{\text{CO}2}$ computed using Eq. 1 successfully replicates the spatial patterns of the simulated fluxes on a global scale (fig. S10). Therefore, Eq. 1 provides a reliable estimation of simulated $F_{\text{CO}2}$, allowing for a robust decomposition of the contributing factors.

### Taylor expansion of the air-sea CO_2_ flux changes

To identify the primary mechanisms driving changes in air-sea CO_2_ flux in the SO under the mitigation scenarios, we used a linear Taylor decomposition of Eq. 1 to analyze flux changes on the basis of key parameters (i.e., wind and $\mathrm{dP}{\text{co}}_{2}$)

$$\underset{\text{Air}-\text{sea}\;CO_{2}\;\text{flux changes}}{\underbrace{∆F_{\text{CO}2}}}\approx \underset{\text{Wind}-\text{driven contribution}}{\underbrace{∆k\alpha \times \bar{\mathrm{dP}{\text{co}}_{2}}}}+\underset{\mathrm{dP}{\text{co}}_{2}-\text{driven contribution}}{\underbrace{\bar{k\alpha }\times ∆\mathrm{dP}{\text{co}}_{2}}}$$ (2)

where ∆ indicates changes relative to the initial value from the reference year 2001, which is represented by the overbar. The right-hand-side terms describe the contribution to $∆F_{\text{CO}2}$ of changes in gas transfer and solubility and the changes in the ocean-atmosphere CO_2_ partial pressure difference. Note that temperature-driven changes in the gas transfer velocity (k) and CO_2_ solubility (α) effectively offset each other because an increase in temperature enhances k while reducing α (*83*–*85*). Consequently, the ∆kα is predominantly driven by wind speed changes.

### Contribution decomposition of ${P\text{co}}_{2}^{\text{ocn}}$ changes

To understand the factors driving changes in ocean CO_2_ partial pressure (${P\text{co}}_{2}^{\text{ocn}}$), we calculated a first-order Taylor series expansion of ${P\text{co}}_{2}^{\text{ocn}}$ as a function of SST, surface DIC, surface total ALK, and sea surface salinity (*48*). In this study, to separate the influence of FW flux, we adopt normalized DIC $\left(\text{sDIC}=S_{\text{ref}}/S\cdot \text{DIC}\right)$ and ALK ($\text{sALK}=S_{\text{ref}}/S\cdot \text{ALK}$) with reference salinity $S_{\text{ref}}$ [35 practical salinity units (psu)]. Hence, the contributions to the ${P\text{co}}_{2}^{\text{ocn}}$ changes read (*49*, *86*, *87*)

$$∆{P\text{co}}_{2}^{\text{ocn}}=\frac{\partial {P\text{co}}_{2}^{\text{ocn}}}{\mathrm{\partial SST}}∆\text{SST}+\frac{\partial {P\text{co}}_{2}^{\text{ocn}}}{\mathrm{\partial DIC}}∆\text{sDIC}+\frac{\partial {P\text{co}}_{2}^{\text{ocn}}}{\mathrm{\partial ALK}}∆\text{sALK}+\frac{\partial {P\text{co}}_{2}^{\text{ocn}}}{\mathrm{\partial FW}}∆\text{FW}$$ (3)

where ∆ indicates the response to the changes relative to the initial value. We use PyCO2SYS to compute the sensitivities of ${P\text{co}}_{2}^{\text{ocn}}$ to DIC and ALK (*88*). Furthermore, we repeat this decomposition with the deviations from the global mean to calculate the regional differences.

## Supplementary Materials

### This PDF file includes:

Supplementary Materials and Methods Table S1 Figs. S1 to S10 References
