# Supplementary material for: Enhanced carbon loss in the Southern Ocean under mitigation scenarios
Source: Sci Adv. 2026 Sep 2;12(36):eaee9228. doi: 10.1126/sciadv.aee9228 (PMC13537242; doi:10.1126/sciadv.aee9228)
Supplement: Supplementary file 1 [file sciadv.aee9228_sm.pdf]

Science Advances
AAAS

Supplementary Materials for

Enhanced carbon loss in the Southern Ocean under mitigation scenarios

Huiji Lee *et al.*

Corresponding author: Jong-Seong Kug, jskug1@gmail.com; Yechul Shin, yechul.ycshin@gmail.com

Sci. Adv. **12**, eaee9228 (2026)
DOI: 10.1126/sciadv.aee9228

This PDF file includes:

Supplementary Materials and Methods
Table S1
Figs. S1 to S10
References

Supplementary Materials and Methods

Coupled Model Intercomparison Project phase 6 (CMIP6)

Ensuring consistent representation of the Southern Ocean (SO; $63°S$ and $47°S$) CO_2 outgassing across different models and experimental configurations is crucial. To show its robustness, we analyzed 8 CMIP6 models using the Zero Emissions Commitment Model Intercomparison Project (ZECMIP) scenario (Supplementary Table S1) (71). Each model dataset was regrided to a regular grid of 1° × 1° horizontal resolution.

Text S1. Equations for solubility and gas exchange in the air-sea CO_2 flux.

In equation (1), the solubility term (α)(*2*) for the air-sea CO_2 flux expresses as

$$\alpha = \exp[-162.8301 + 219.2969\left(\frac{100}{T}\right) + 90.9241\ln\left(\frac{T}{100}\right) - 1.47696\left(\frac{T}{100}\right)^2 + S(0.025695 - 0.025225\left(\frac{T}{100}\right) + 0.0049867\left(\frac{T}{100}\right)^2)], \quad (1)$$

where T is sea surface temperature (SST; K), and S is sea surface salinity (SSS; PSU). Also, the term for gas exchange velocity (k)(*82*) is defined by

$$k = 0.251 \times \sqrt{\frac{660}{S_c}} \times (U_{10})^2, \quad (2)$$

where U_{10} is the 10-meter wind speed (m/s). The Schmidt number, S_c, depends on SST (T; ^{o}C) and is expressed as $S_c = 2116.8 + T(-136.25 + T(4.7353 + T(-0.092307 + 0.0007555 * T)))(82)$.

Table S1. Description of CMIP6 models used in this study.

Model names	Institution
ACCESS-ESM1-5 (8*9*)	CSIRO and ARCCSS, Australia
CESM2 (74)	NCAR, USA
CanESM5 (*90*)	CCCma, Canada
MPI-ESM1-2-LR (91)	MPI-M, Germany
MIROC-ES2L (92)	MRI, Japan
NorESM2-LM (*93*, 94)	NORCE, Norway
GISS-E2-1-G-CC (95)	NASA/GISS, USA
UKESM1-0-LL (96)	MOHC, UK

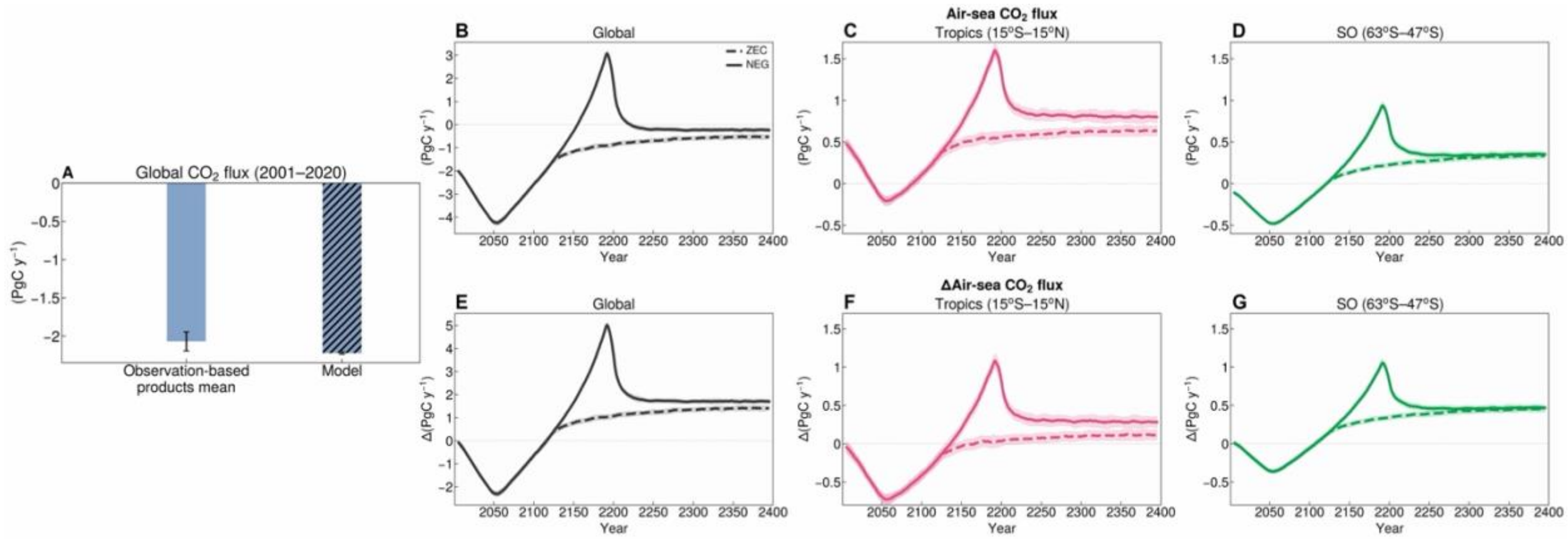

Fig. S1. Area-integrated ocean CO_2 uptake under the mitigation experiments. (A) The bar graph of the global mean CO_2 uptake averaged over 2001-2020, comparing the observation-based air-sea CO_2 flux data mean (non-hatched bar) with the model simulation (hatched bar). Regional time series of the area-integrated air-sea CO_2 flux for global mean (black/grey lines), tropics (pink lines, 15ºS-15ºN), and Southern Ocean (SO; green lines, 63ºS-47ºS) **(B-D)** in the absolute values and **(E-G)** changes relative to the initial 5 years mean. Solid lines and dashed lines represent the NEG and ZEC experiments, respectively (shading shows inter-ensemble spread $\pm 1\ std$).

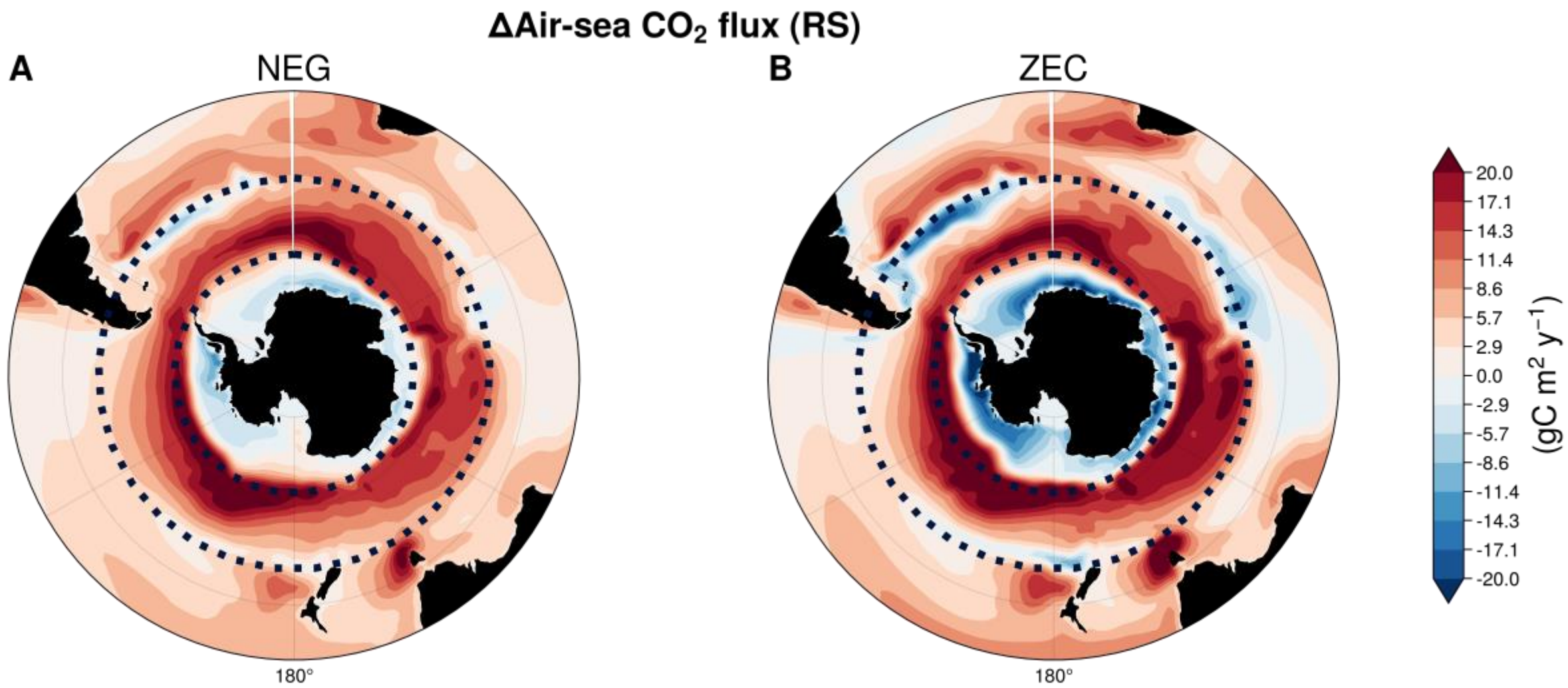

Fig. S2. Air-sea CO_2 flux changes over the Southern Ocean (south of 30°S). Spatial pattern of the air-sea CO_2 changes over the south of 30°S for the **(A)** negative emission (NEG) and **(B)** net-zero emission (ZEC) experiments. The dashed black lines indicate the SO, which are defined in this study between 63°S and 47°S. The anomaly is calculated by comparing the restoring period (RS; years 2300 to 2400) to the initial 5 years.

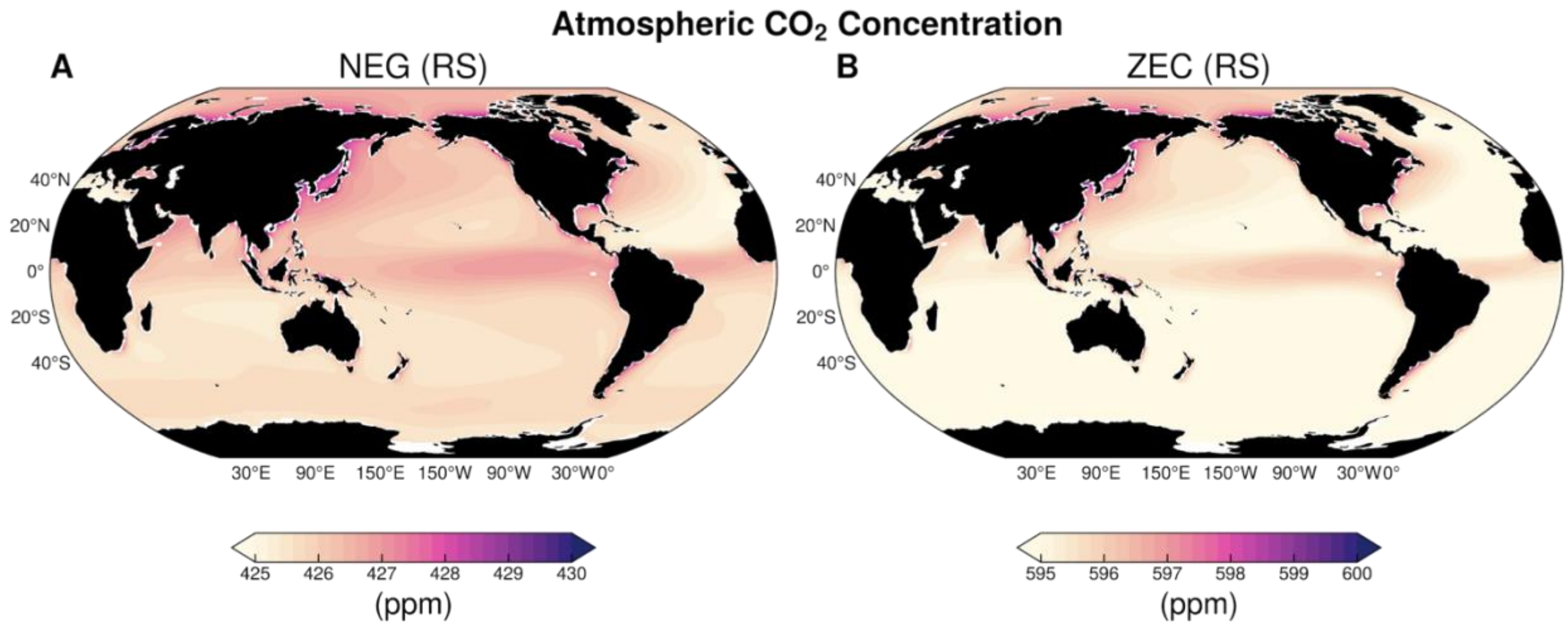

Fig. S3. Spatial patterns of the atmospheric CO_2 concentration. (A, B) Annual mean atmospheric CO_2 concentration during the restoring period (RS, years 2300 to 2400).

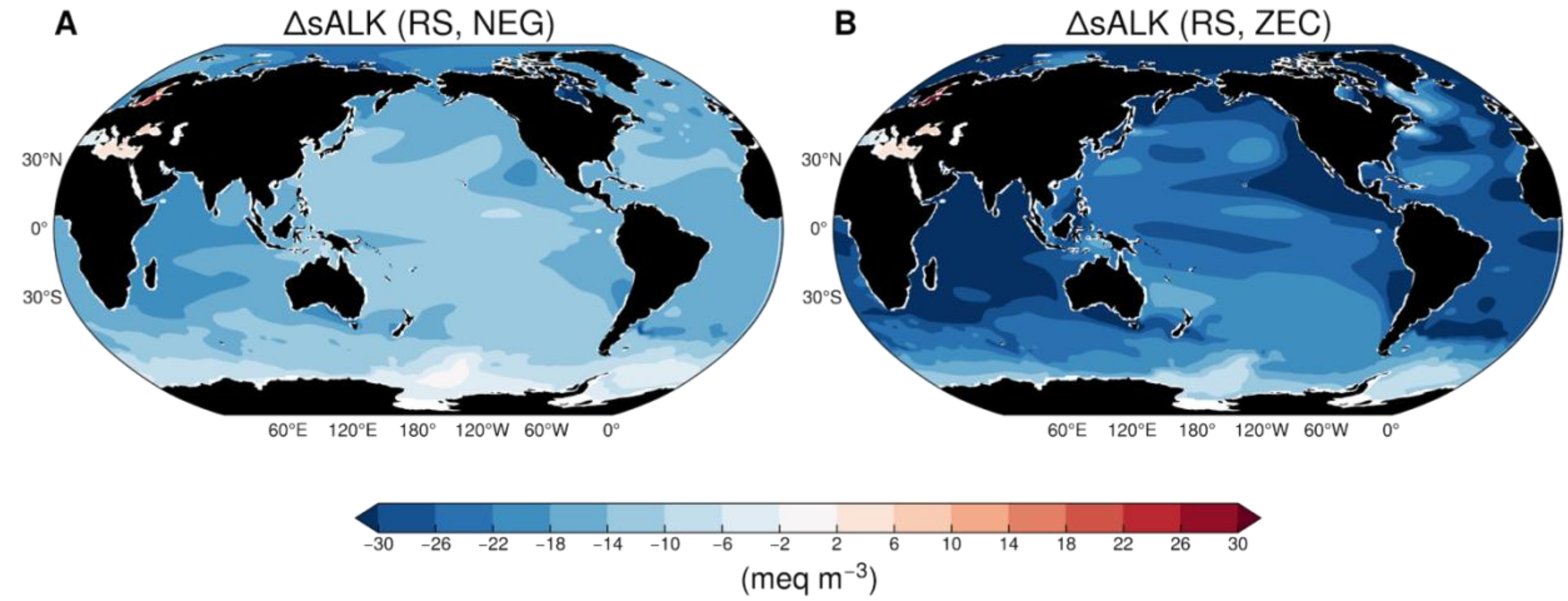

Fig. S4. Spatial patterns of the surface changes of salinity normalized alkalinity (sALK). (A, B) Surface changes in salinity normalized alkalinity between the annual mean of initial years from 2001 to 2005 and the annual mean of the restoring period (RS, years 2300 to 2400). Alkalinity is averaged over the upper ocean (0–100m) and normalized with a standard salinity of 35 psu.

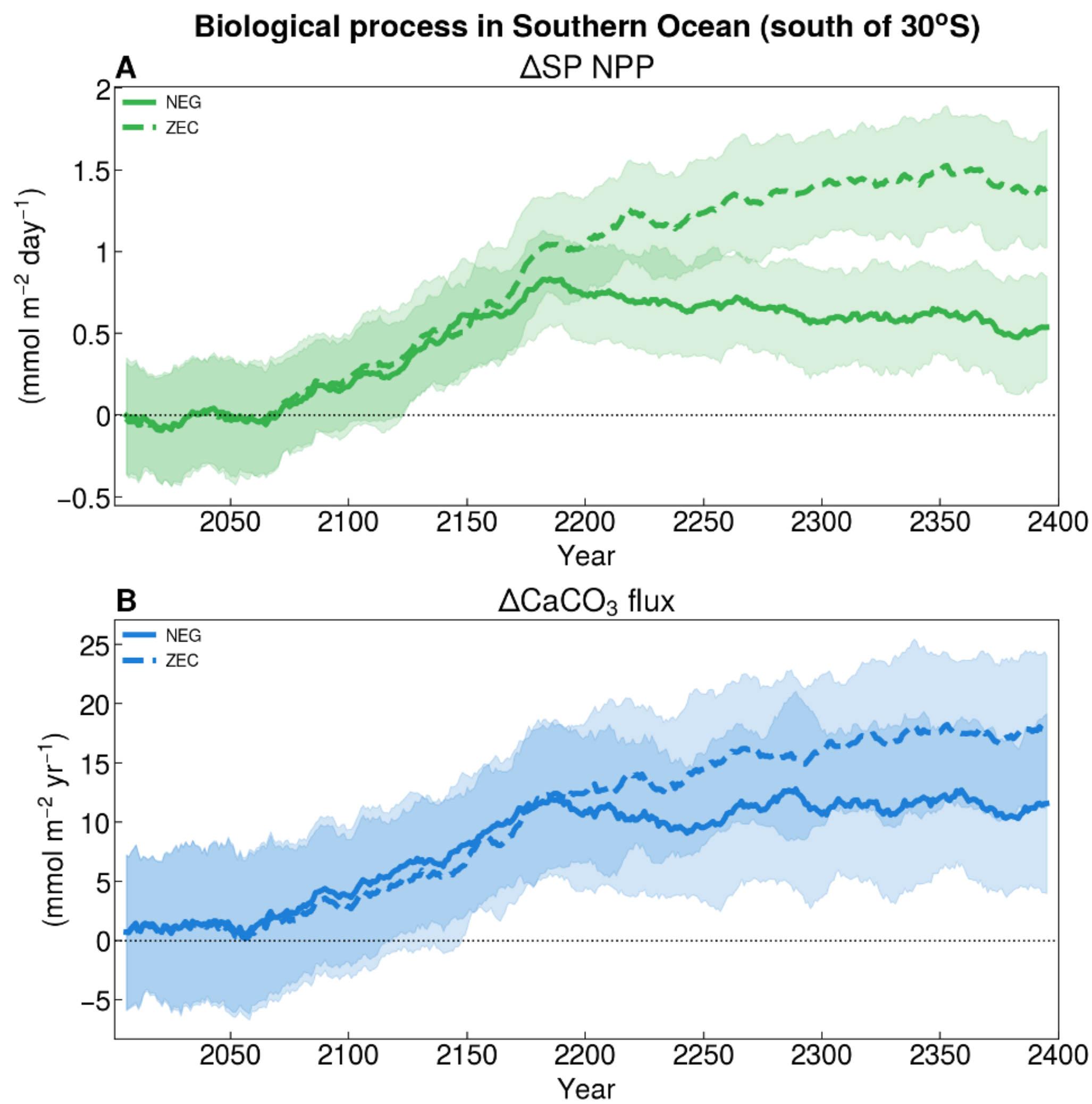

Fig. S5. Biological process changes over the Southern Ocean (south of 30°S). (A) Time series of small phytoplankton Net Primary Production (SP NPP) changes and **(B)** of calcium carbonate ($CaCO_3$) flux changes around the south of 30°S in NEG (solid) and ZEC (dashed). Shading indicates to inter-ensemble spread $\pm 1\ std$.

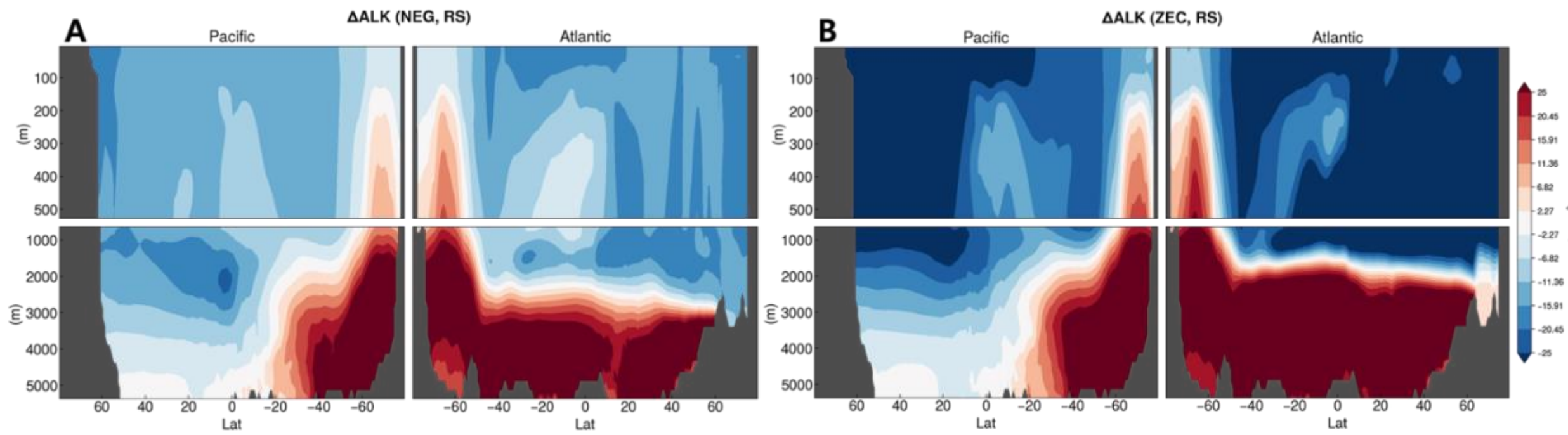

Fig. S6. Vertical distributions of the salinity normalized alkalinity. Vertical distribution of the ALK changes in the **(A)** NEG and **(B)** ZEC experiments. The anomaly is calculated by comparing the restoring period (RS; years 2300 to 2400) to the initial 10 years (2001-2010). ALK is normalized by salinity (the standard salinity is 35 psu).

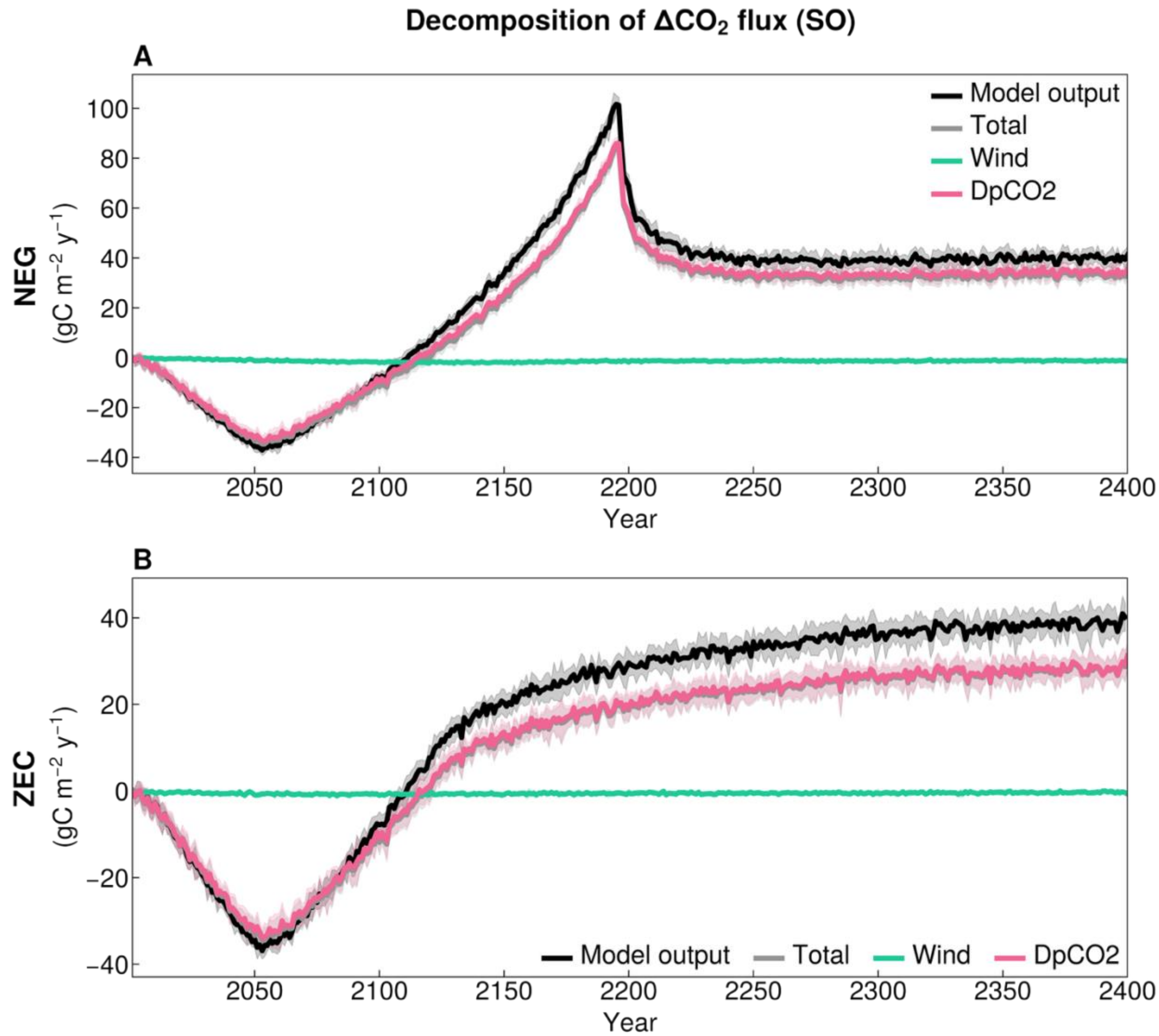

Fig. S7. Decomposition of the air-sea CO_2 flux changes in the Southern Ocean. Temporal evolution of decomposition of CO_2 flux changes (ΔCO_2 flux) in the **(A)** NEG and **(B)** ZEC experiments, relative to an initial year 2001. The colored lines represent contributions from wind-driven contribution (green) and $dpCO_2$-driven contribution (pink), while the total contribution (grey) corresponds to the sum of these components. All values of colored lines represent the ensemble mean and shading is the inter-ensemble spread ($\pm 1\ std$). It is smoothed with an 11-year running mean.

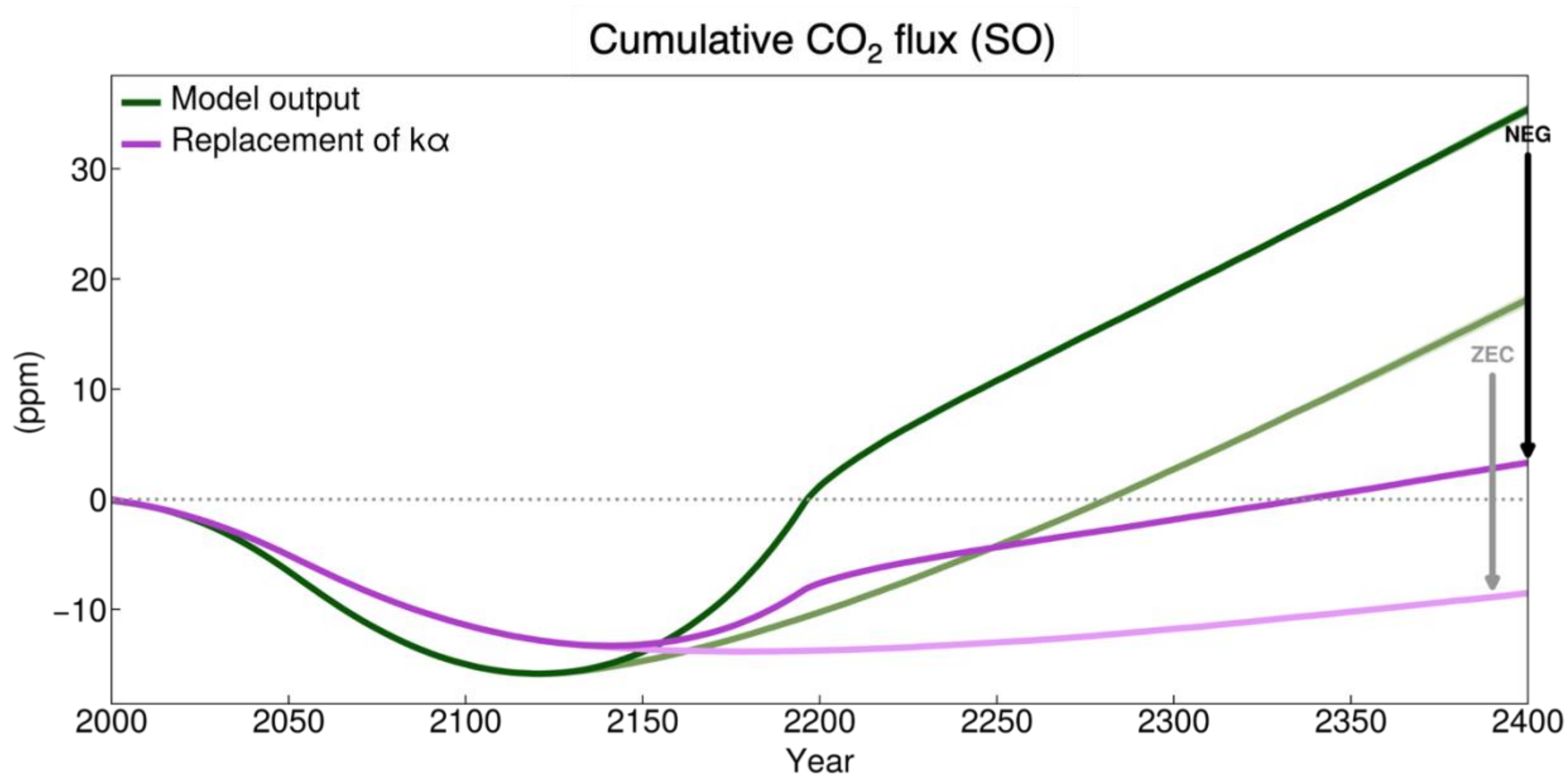

Fig. S8. Assessing the sensitivity of $k\alpha$ in the Southern Ocean cumulative CO_2 flux. Time evolution of cumulative CO_2 flux in the Southern Ocean (SO; 63ºS-47ºS), converted from Pg C to ppm. Green lines indicate the original model-simulated cumulative CO_2 flux: dark green line for the NEG simulation and the light green for the ZEC simulation. Purple lines depict the reconstructed cumulative CO_2 flux using adjusted $k\alpha$ to the global mean: dark purple for the NEG and the light purple for the ZEC. All values of colored lines represent the ensemble mean and shading shows the uncertainty of the projections calculated by the inter-ensemble spread ($\pm 1\ std$).

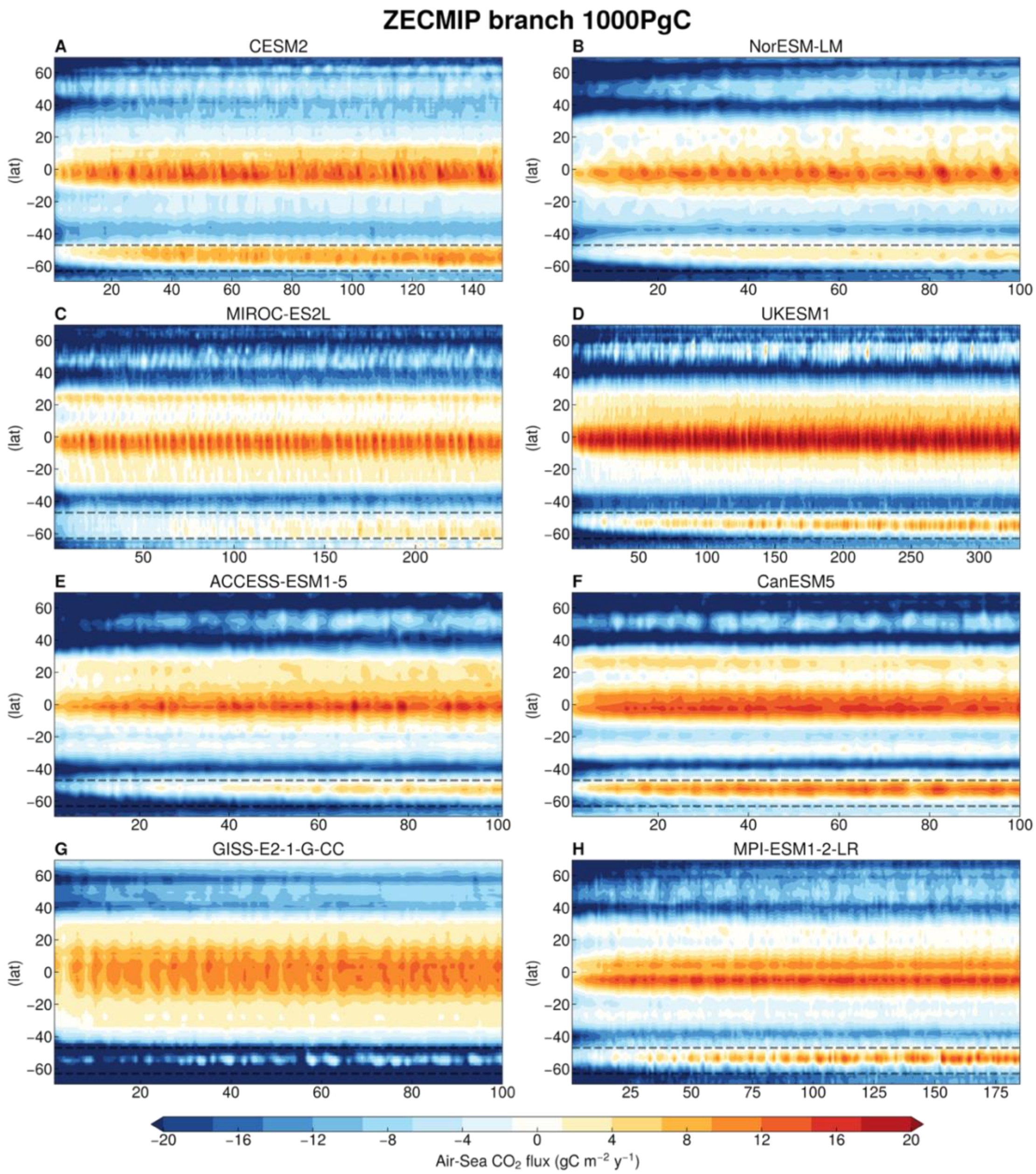

Fig. S9. Air-sea CO_2 flux response under the 1000 Pg C Zero Emissions Commitment Model Intercomparison Project (ZECMIP) scenario. (A-H) Air-sea CO_2 flux from CMIP6 multi model ZECMIP simulations, shown from the year following the cessation of emissions. Note that the time range varies across experiments. Dashed horizontal lines show the Southern Ocean region (SO; 63ºS-47ºS). Negative (positive) air-sea CO_2 flux indicates oceanic CO_2 uptake (outgassing).

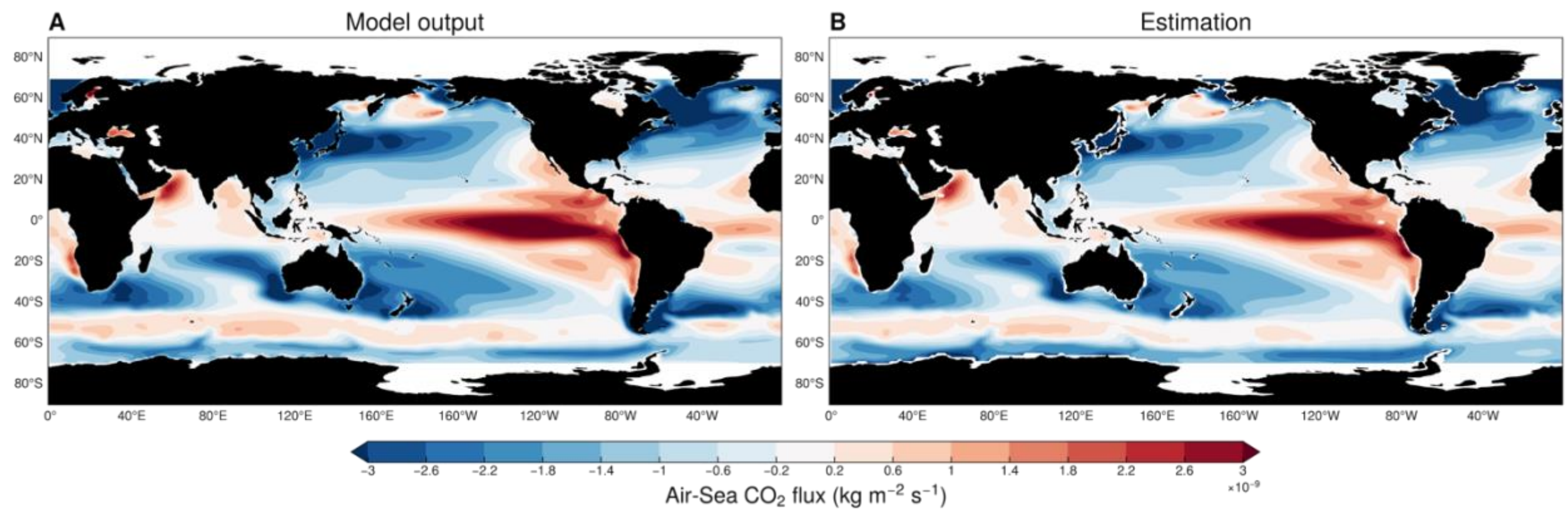

Fig. S10. Estimation of the air-sea CO_2 flux. Spatial distribution of air-sea CO_2 flux in the initial year 2001 from the **(A)** model output and **(B)** the reconstructed estimate. In this study, the flux estimation is limited to the global ocean between 65°S and 65°N, excluding sea-ice-covered regions. Negative (positive) air-sea CO_2 flux indicates oceanic CO_2 uptake (outgassing).

REFERENCES

1. R. K. Pachauri, M. R. Allen, V. R. Barros, J. Broome, W. Cramer, R. Christ, J. A. Church, L. Clarke, Q. Dahe, P. Dasgupta, N. K. Dubash, O. Edenhofer, I. Elgizouli, C. B. Field, P. Forster, P. Friedlingstein, J. Fuglestvedt, L. Gomez-Echeverri, S. Hallegatte, G. Hegerl, M. Howden, K. Jiang, B. Jimenez Cisneroz, V. Kattsov, H. Lee, K. J. Mach, J. Marotzke, M. D. Mastrandrea, L. Meyer, J. Minx, Y. Mulugetta, K. O'Brien, M. Oppenheimer, J. J. Pereira, R. Pichs-Madruga, G.-K. Plattner, H.-O. Pörtner, S. B. Power, B. Preston, N. H. Ravindranath, A. Reisinger, K. Riahi, M. Rusticucci, R. Scholes, K. Seyboth, Y. Sokona, R. Stavins, T. F. Stocker, P. Tschakert, D. van Vuuren, J.-P. van Ypserle, *Climate Change 2014: Synthesis Report. Contribution of Working Groups I, II and III to the Fifth Assessment Report of the Intergovernmental Panel on Climate Change* (IPCC, Geneva, Switzerland, 2014); https://epic.awi.de/id/eprint/37530/.

2. M. R. Allen, D. J. Frame, C. Huntingford, C. D. Jones, J. A. Lowe, M. Meinshausen, N. Meinshausen, Warming caused by cumulative carbon emissions towards the trillionth tonne. *Nature* **458**, 1163–1166 (2009).

3. T. A. Carleton, S. M. Hsiang, Social and economic impacts of climate. *Science* **353**, aad9837 (2016).

4. H. D. Matthews, N. P. Gillett, P. A. Stott, K. Zickfeld, The proportionality of global warming to cumulative carbon emissions. *Nature* **459**, 829–832 (2009).

5. United Nations Framework on Climate Change (UNFCCC), Adoption of the Paris Agreement, 21st Conference of the Parties (2015); http://unfccc.int/resource/docs/2015/cop21/eng/l09r01.pdf.

6. T. Gasser, C. Guivarch, K. Tachiiri, C. D. Jones, P. Ciais, Negative emissions physically needed to keep global warming below 2 °C. *Nat. Commun.* **6**, 7958 (2015).

7. E. Kriegler, G. Luderer, N. Bauer, L. Baumstark, S. Fujimori, A. Popp, J. Rogelj, J. Strefler, D. P. van Vuuren, Pathways limiting warming to 1.5°C: A tale of turning around in no time? *Philos. Trans. A Math. Phys. Eng. Sci.* **376**, 20160457 (2018).

8. D. P. Keller, A. Lenton, V. Scott, N. E. Vaughan, N. Bauer, D. Ji, C. D. Jones, B. Kravitz, H. Muri, K. Zickfeld, The Carbon Dioxide Removal Model Intercomparison Project (CDRMIP): Rationale and experimental protocol for CMIP6. *Geosci. Model Dev.* **11**, 1133–1160 (2018).

9. J. Rogelj, M. Schaeffer, P. Friedlingstein, N. P. Gillett, D. P. van Vuuren, K. Riahi, M. Allen, R. Knutti, Differences between carbon budget estimates unravelled. *Nat. Clim. Chang.* **6**, 245–252 (2016).

10. J. Schwinger, A. Asaadi, N. J. Steinert, H. Lee, Emit now, mitigate later? Earth system reversibility under overshoots of different magnitudes and durations. *Earth Syst. Dynam.* **13**, 1641–1665 (2022).

11. O. Boucher, P. R. Halloran, E. J. Burke, M. Doutriaux-Boucher, C. D. Jones, J. Lowe, M. A. Ringer, E. Robertson, P. Wu, Reversibility in an Earth System model in response to CO_2 concentration changes. *Environ. Res. Lett.* **7**, 024013 (2012).

12. S.-K. Kim, J. Shin, S.-I. An, H.-J. Kim, N. Im, S.-P. Xie, J.-S. Kug, S.-W. Yeh, Widespread irreversible changes in surface temperature and precipitation in response to CO_2 forcing. *Nat. Clim. Chang.* **12**, 834–840 (2022).

13. J.-S. Kug, J.-H. Oh, S.-I. An, S.-W. Yeh, S.-K. Min, S.-W. Son, J. Kam, Y.-G. Ham, J. Shin, Hysteresis of the intertropical convergence zone to CO_2 forcing. *Nat. Clim. Chang.* **12**, 47–53 (2022).

14. T. Iwakiri, J.-S. Kug, F.-F. Jin, S. Zhao, S.-I. An, G.-I. Kim, D. Park, Abrupt shift of El Niño periodicity under CO_2 mitigation. *Proc. Natl. Acad. Sci.U.S.A.* **122**, e2426048122 (2025).

15. A. Jeltsch-Thömmes, T. F. Stocker, F. Joos, Hysteresis of the Earth system under positive and negative CO_2 emissions. *Environ. Res. Lett.* **15**, 124026 (2020).

16. C. Liu, S.-I. An, F.-F. Jin, J. Shin, J.-S. Kug, W. Zhang, M. F. Stuecker, X. Yuan, A. Xue, X. Geng, S.-K. Kim, Hysteresis of the El Niño–Southern Oscillation to CO_2 forcing. *Sci. Adv.* **9**, eadh8442 (2023).

17. E. A. Monteiro, D. P. Keller, J. R. Christian, J. G. John, M. Kawamiya, R. Séférian, J. Tjiputra, A. Wiltshire, A. Oschlies, Degrees of reversibility of ocean deoxygenation in an atmospheric carbon dioxide removal scenario. *Environ. Res. Lett.* **20**, 084051 (2025).

18. D. Couespel, J. Tjiputra, What goes in must come out: The oceanic outgassing of anthropogenic carbon. *Environ. Res. Lett.* **19**, 014086 (2024).

19. L. Bertini, J. Tjiputra, Biogeochemical timescales of climate change onset and recovery in the North Atlantic interior under rapid atmospheric CO_2 forcing. *J. Geophys. Res. Oceans* **127**, e2021JC017929 (2022).

20. D.-G. Lee, E. Y. Kwon, J. Kam, J.-S. Kug, Irreversible phytoplankton community shifts over Subpolar North Atlantic in response to CO_2 forcing. *Earth Syst. Dynam.* **16**, 2101–2111 (2025).

21. N. Gruber, D. Clement, B. R. Carter, R. A. Feely, S. Van Heuven, M. Hoppema, M. Ishii, R. M. Key, A. Kozyr, S. K. Lauvset, C. L. Monaco, J. T. Mathis, A. Murata, A. Olsen, F. F. Perez, C. L. Sabine, T. Tanhua, R. Wanninkhof, The oceanic sink for anthropogenic CO_2 from 1994 to 2007. *Science* **363**, 1193–1199 (2019).

22. N. Gruber, D. C. E. Bakker, T. DeVries, L. Gregor, J. Hauck, P. Landschützer, G. A. McKinley, J. D. Müller, Trends and variability in the ocean carbon sink. *Nat. Rev. Earth Environ.* **4**, 119–134 (2023).

23. P. Friedlingstein, M. O'Sullivan, M. W. Jones, R. M. Andrew, J. Hauck, P. Landschützer, C. Le Quéré, H. Li, I. T. Luijkx, A. Olsen, G. P. Peters, W. Peters, J. Pongratz, C. Schwingshackl, S. Sitch, J. G. Canadell, P. Ciais, R. B. Jackson, S. R. Alin, A. Arneth, V. Arora, N. R. Bates, M. Becker, N. Bellouin, C. F. Berghoff, H. C. Bittig, L. Bopp, P. Cadule, K. Campbell, M. A. Chamberlain, N. Chandra, F. Chevallier, L. P. Chini, T. Colligan, J. Decayeux, L. M. Djeutchouang, X. Dou, C. Duran Rojas, K. Enyo, W. Evans, A. R. Fay, R. A. Feely, D. J. Ford, A. Foster, T. Gasser, M. Gehlen, T. Gkritzalis, G. Grassi, L. Gregor, N. Gruber, Ö. Gürses, I. Harris, M. Hefner, J. Heinke, G. C. Hurtt, Y. Iida, T. Ilyina, A. R. Jacobson, A. K. Jain, T. Jarníková, A. Jersild, F. Jiang, Z. Jin, E. Kato, R. F. Keeling, K. Klein Goldewijk, J. Knauer, J. I. Korsbakken, X. Lan, S. K. Lauvset, N. Lefèvre, Z. Liu, J. Liu, L.

Ma, S. Maksyutov, G. Marland, N. Mayot, P. C. McGuire, N. Metzl, N. M. Monacci, E. J. Morgan, S.-I. Nakaoka, C. Neill, Y. Niwa, T. Nützel, L. Olivier, T. Ono, P. I. Palmer, D. Pierrot, Z. Qin, L. Resplandy, A. Roobaert, T. M. Rosan, C. Rödenbeck, J. Schwinger, T. L. Smallman, S. M. Smith, R. Sospedra-Alfonso, T. Steinhoff, Q. Sun, A. J. Sutton, R. Séférian, S. Takao, H. Tatebe, H. Tian, B. Tilbrook, O. Torres, E. Tourigny, H. Tsujino, F. Tubiello, G. van der Werf, R. Wanninkhof, X. Wang, D. Yang, X. Yang, Z. Yu, W. Yuan, X. Yue, S. Zaehle, N. Zeng, J. Zeng, Global Carbon Budget 2024. *Earth Syst. Sci. Data* **17**, 965–1039 (2025).

24. N. Gruber, P. Landschützer, N. S. Lovenduski, The variable Southern Ocean carbon sink. *Ann. Rev. Mar. Sci.* **11**, 159–186 (2019).

25. J. Hauck, L. Gregor, C. Nissen, L. Patara, M. Hague, P. Mongwe, S. Bushinsky, S. C. Doney, N. Gruber, C. Le Quéré, M. Manizza, M. Mazloff, P. M. S. Monteiro, J. Terhaar, The Southern Ocean carbon cycle 1985–2018: Mean, seasonal cycle, trends, and storage. *Global Biogeochem. Cycles* **37**, e2023GB007848 (2023).

26. Y. Dong, D. C. E. Bakker, T. G. Bell, M. Yang, P. Landschützer, J. Hauck, C. Rödenbeck, V. Kitidis, S. M. Bushinsky, P. S. Liss, Direct observational evidence of strong CO_2 uptake in the Southern Ocean. *Sci. Adv.* **10**, eadn5781 (2024).

27. A. Kessler, J. Tjiputra, The Southern Ocean as a constraint to reduce uncertainty in future ocean carbon sinks. *Earth Syst. Dynam.* **7**, 295–312 (2016).

28. T. Bourgeois, N. Goris, J. Schwinger, J. F. Tjiputra, Stratification constrains future heat and carbon uptake in the Southern Ocean between 30°S and 55°S. *Nat. Commun.* **13**, 340 (2022).

29. J. Terhaar, T. L. Frölicher, F. Joos, Southern Ocean anthropogenic carbon sink constrained by sea surface salinity. *Sci. Adv.* **7**, eabd5964 (2021).

30. M. O. Chikamoto, P. DiNezio, Multi-century changes in the ocean carbon cycle controlled by the tropical oceans and the Southern Ocean. *Global Biogeochem. Cycles* **35**, e2021GB007090 (2021).

31. R. Bernardello, I. Marinov, J. B. Palter, J. L. Sarmiento, E. D. Galbraith, R. D. Slater, Response of the ocean natural carbon storage to projected twenty-first-century climate change. *J. Climate* **27**, 2033–2053 (2014).

32. T. DeVries, The ocean carbon cycle. *Annu. Rev. Env. Resour.* **47**, 317–341 (2022).

33. H. Lee, K.-M. Noh, J.-H. Oh, S.-W. Park, Y. Shin, J.-S. Kug, Emergence of an oceanic CO_2 uptake hole under global warming. *Nat. Commun.* **16**, 3199 (2025).

34. S. M. Ridge, G. A. McKinley, Ocean carbon uptake under aggressive emission mitigation. *Biogeosciences* **18**, 2711–2725 (2021).

35. P. Mongwe, L. Gregor, J. Tjiputra, J. Hauck, T. Ito, C. Danek, M. Vichi, S. Thomalla, P. M. S. Monteiro, Projected poleward migration of the Southern Ocean CO_2 sink region under high emissions. *Commun. Earth Environ.* **5**, 232 (2024).

36. J. F. Tjiputra, K. Assmann, C. Heinze, Anthropogenic carbon dynamics in the changing ocean. *Ocean Sci.* **6**, 605–614 (2010).

37. K. Power, F. D. A. Oliveira Matos, Q. Zhang, Late Pliocene ice sheets as an analogue for future climate: A sensitivity study of the polar Southern Hemisphere. *Earth Syst. Dynam.* **16**, 1845–1863 (2025).

38. R. G. Williams, P. Ceppi, V. Roussenov, A. Katavouta, A. J. S. Meijers, The role of the Southern Ocean in the global climate response to carbon emissions. *Philos. Trans. A Math. Phys. Eng. Sci.* **381**, 20220062 (2023).

39. J. B. Kajtar, A. Santoso, M. Collins, A. S. Taschetto, M. H. England, L. M. Frankcombe, CMIP5 intermodel relationships in the baseline Southern Ocean climate system and with future projections. *Earth's Future* **9**, e2020EF001873 (2021).

40. P. J. Roldán-Gómez, P. Ortega, M. G. Donat, Contribution of meridional overturning circulation and sea ice changes to large-scale temperature asymmetries in CMIP6 overshoot scenarios. *Ocean Sci.* **21**, 2283–2303 (2025).

41. J.-H. Oh, J.-S. Kug, S.-I. An, F.-F. Jin, M. J. McPhaden, J. Shin, Emergent climate change patterns originating from deep ocean warming in climate mitigation scenarios. *Nat. Clim. Chang.* **14**, 260–266 (2024).

42. S. Wang, Y. Shin, J.-H. Oh, H. Kim, J.-S. Kug, Critical role of low cloud feedback in irreversible sea level rise. *Nat. Commun.* **17**, 6292 (2026).

43. C. D. Koven, V. K. Arora, P. Cadule, R. A. Fisher, C. D. Jones, D. M. Lawrence, J. Lewis, K. Lindsay, S. Mathesius, M. Meinshausen, M. Mills, Z. Nicholls, B. M. Sanderson, R. Séférian, N. C. Swart, W. R. Wieder, K. Zickfeld, Multi-century dynamics of the climate and carbon cycle under both high and net negative emissions scenarios. *Earth Syst. Dynam.* **13**, 885–909 (2022).

44. W. Yao, L. Cao, X. Jin, Simulated responses of the ocean carbon cycle to different rates of atmospheric CO_2 removal. *J. Geophys. Res. Oceans* **130**, e2024JC022115 (2025).

45. A. J. Watson, U. Schuster, J. D. Shutler, T. Holding, I. G. C. Ashton, P. Landschützer, D. K. Woolf, L. Goddijn-Murphy, Revised estimates of ocean-atmosphere CO_2 flux are consistent with ocean carbon inventory. *Nat. Commun.* **11**, 4422 (2020).

46. R. Wanninkhof, G.-H. Park, T. Takahashi, C. Sweeney, R. Feely, Y. Nojiri, N. Gruber, S. C. Doney, G. A. McKinley, A. Lenton, Global ocean carbon uptake: Magnitude, variability and trends. *Biogeosciences* **10**, 1983–2000 (2013).

47. T. Takahashi, S. C. Sutherland, C. Sweeney, A. Poisson, N. Metzl, B. Tilbrook, N. Bates, R. Wanninkhof, R. A. Feely, C. Sabine, Global sea–air CO_2 flux based on climatological surface ocean pCO_2, and seasonal biological and temperature effects. *Deep-Sea Res. II Top. Stud. Oceanogr.* **49**, 1601–1622 (2002).

48. T. Takahashi, S. C. Sutherland, R. Wanninkhof, C. Sweeney, R. A. Feely, D. W. Chipman, B. Hales, G. Friederich, F. Chavez, C. Sabine, J. Olafsson, Y. Nojiri, Climatological mean and decadal change in surface ocean pCO_2, and net sea–air CO_2 flux over the global oceans. *Deep-Sea Res. II Top. Stud. Oceanogr.* **56**, 554–577 (2009).

49. P. Landschützer, N. Gruber, D. C. Bakker, I. Stemmler, K. D. Six, Strengthening seasonal marine CO_2 variations due to increasing atmospheric CO_2. *Nat. Clim. Chang.* **8**, 146–150 (2018).

50. I. Frenger, S. Frey, A. Oschlies, J. Getzlaff, T. Martin, W. Koeve, Southern Ocean heat burp in a cooling world. *AGU Adv.* **6**, e2025AV001700 (2025).

51. P. Ceppi, P. Nowack, Observational evidence that cloud feedback amplifies global warming. *Proc. Natl. Acad. Sci. U.S.A.* **118**, e2026290118 (2021).

52. T. Ogura, M. J. Webb, A. P. Lock, Positive low cloud feedback primarily caused by increasing longwave radiation from the sea surface in two versions of a climate model. *Geophys. Res. Lett.* **50**, e2023GL104786 (2023).

53. J. A. Curry, J. L. Schramm, E. E. Ebert, Sea ice-albedo climate feedback mechanism. *J. Clim.* **8**, 240–247 (1995).

54. J. A. Screen, I. Simmonds, The central role of diminishing sea ice in recent Arctic temperature amplification. *Nature* **464**, 1334–1337 (2010).

55. T. Toyoda, T. Aoki, M. Niwano, T. Tanikawa, L. S. Urakawa, H. Tsujino, H. Nakano, K. Sakamoto, N. Hirose, G. Yamanaka, Impact of observation-based snow albedo parameterization on global ocean simulation results. *Polar Sci.* **24**, 100521 (2020).

56. S. C. Doney, Plankton in a warmer world. *Nature* **444**, 695–696 (2006).

57. M. Winder, U. Sommer, Phytoplankton response to a changing climate. *Hydrobiologia* **698**, 5–16 (2012).

58. K. M. Krumhardt, M. C. Long, K. Lindsay, M. N. Levy, Southern Ocean calcification controls the global distribution of alkalinity. *Global Biogeochem. Cycles* **34**, e2020GB006727 (2020).

59. F. W. Primeau, M. Holzer, T. DeVries, Southern Ocean nutrient trapping and the efficiency of the biological pump. *J. Geophys. Res. Oceans* **118**, 2547–2564 (2013).

60. L. Kwiatkowski, O. Torres, L. Bopp, O. Aumont, M. Chamberlain, J. R. Christian, J. P. Dunne, M. Gehlen, T. Ilyina, J. G. John, A. Lenton, H. Li, N. S. Lovenduski, J. C. Orr, J. Palmieri, Y. Santana-Falcón, J. Schwinger, R. Séférian, C. A. Stock, A. Tagliabue, Y. Takano, J. Tjiputra, K. Toyama, H. Tsujino, M. Watanabe, A. Yamamoto, A. Yool, T. Ziehn, Twenty-first century ocean warming, acidification, deoxygenation, and upper-ocean nutrient and primary production decline from CMIP6 model projections. *Biogeosciences* **17**, 3439–3470 (2020).

61. M. O. Chikamoto, P. DiNezio, N. Lovenduski, Long-term slowdown of ocean carbon uptake by alkalinity dynamics. *Geophys. Res. Lett.* **50**, e2022GL101954 (2023).

62. T. Takahashi, R. A. Feely, R. F. Weiss, R. H. Wanninkhof, D. W. Chipman, S. C. Sutherland, T. T. Takahashi, Global air-sea flux of CO_2: An estimate based on measurements of sea–air pCO_2 difference. *Proc. Natl. Acad. Sci. U.S.A.* **94**, 8292–8299 (1997).

63. R. Wanninkhof, Relationship between wind speed and gas exchange over the ocean. *J. Geophys. Res. Oceans* **97**, 7373–7382 (1992).

64. A. Planchat, L. Bopp, L. Kwiatkowski, O. Torres, The carbonate pump feedback on alkalinity and the carbon cycle in the 21st century and beyond. *Earth Syst. Dynam.* **15**, 565–588 (2024).

65. C. Hinrichs, P. Köhler, C. Völker, J. Hauck, Alkalinity biases in CMIP6 Earth system models and implications for simulated CO_2 drawdown via artificial alkalinity enhancement. *Biogeosciences* **20**, 3717–3735 (2023).

66. M. C. Long, J. K. Moore, K. Lindsay, M. Levy, S. C. Doney, J. Y. Luo, K. M. Krumhardt, R. T. Letscher, M. Grover, Z. T. Sylvester, Simulations with the marine biogeochemistry library (MARBL). *J. Adv. Model. Earth Syst.* **13**, e2021MS002647 (2021).

67. W. Cai, L. Gao, Y. Luo, X. Li, X. Zheng, X. Zhang, X. Cheng, F. Jia, A. Purich, A. Santoso, Y. Du, D. M. Holland, J.-R. Shi, B. Xiang, S.-P. Xie, Southern Ocean warming and its climatic impacts. *Sci. Bull.* **68**, 946–960 (2023).

68. S.-J. Shin, S.-W. Yeh, S.-I. An, N. Keenlyside, S.-P. Xie, J.-H. Park, Southern Ocean control of 2°C global warming in climate models. *Earth's Future* **11**, e2022EF003212 (2023).

69. J.-B. Sallée, Southern Ocean warming. *Oceanography* **31**, 52–62 (2018).

70. L. J. Cassidy, A. D. King, J. R. Brown, A. H. MacDougall, T. Ziehn, S.-K. Min, C. D. Jones, Regional temperature extremes and vulnerability under net zero CO_2 emissions. *Environ. Res. Lett.* **19**, 014051 (2023).

71. C. D. Jones, T. L. Frölicher, C. Koven, A. H. MacDougall, H. D. Matthews, K. Zickfeld, J. Rogelj, K. B. Tokarska, N. P. Gillett, T. Ilyina, M. Meinshausen, N. Mengis, R. Séférian, M. Eby, F. A. Burger, The Zero Emissions Commitment Model Intercomparison Project (ZECMIP) contribution to C4MIP: Quantifying committed climate changes following zero carbon emissions. *Geosci. Model Dev.* **12**, 4375–4385 (2019).

72. T. J. Bracegirdle, E. Shuckburgh, J.-B. Sallee, Z. Wang, A. J. S. Meijers, N. Bruneau, T. Phillips, L. J. Wilcox, Assessment of surface winds over the Atlantic, Indian, and Pacific Ocean sectors of the Southern Ocean in CMIP5 models: Historical bias, forcing response, and state dependence. *J. Geophys. Res. Atmos.* **118**, 547–562 (2013).

73. T. L. Frölicher, J. L. Sarmiento, D. J. Paynter, J. P. Dunne, J. P. Krasting, M. Winton, Dominance of the Southern Ocean in anthropogenic carbon and heat uptake in CMIP5 models. *J. Clim.* **28**, 862–886 (2015).

74. G. Danabasoglu, J.-F. Lamarque, J. Bacmeister, D. A. Bailey, A. K. DuVivier, J. Edwards, L. K. Emmons, J. Fasullo, R. Garcia, A. Gettelman, C. Hannay, M. M. Holland, W. G. Large, P. H. Lauritzen, D. M. Lawrence, J. T. M. Lenaerts, K. Lindsay, W. H. Lipscomb, M. J. Mills, R. Neale, K. W. Oleson, B. Otto-Bliesner, A. S. Phillips, W. Sacks, S. Tilmes, L. van Kampenhout, M. Vertenstein, A. Bertini, J. Dennis, C. Deser, C. Fischer, B. Fox-Kemper, J. E. Kay, D. Kinnison, P. J. Kushner, V. E. Larson, M. C. Long, S. Mickelson, J. K. Moore, E. Nienhouse, L. Polvani, P. J. Rasch, W. G. Strand, The Community Earth System Model Version 2 (CESM2). *J. Adv. Model. Earth Syst.* **12**, e2019MS001916 (2020).

75. R. Smith, P. Jones, P. Briegleb, O. Bryan, G. Danabasoglu, M. Dennis, J. Dukowicz, C. Eden, B. Fox-Kemper, R. Gent, M. Hecht, S. Jayne, M. Jochum, G. Large, K. Lindsay, M. Maltrud, J. Norton, L. Peacock, M. Vertenstein, S. Yeager, The Parallel Ocean Program (POP) reference manual: Ocean component of the Community Climate System Model (CCSM) (NCAR Report, 2010).

76. D. A. Bailey, M. M. Holland, A. K. DuVivier, E. C. Hunke, A. K. Turner, Impact of a new sea ice thermodynamic formulation in the CESM2 sea ice component. *J. Adv. Model. Earth Syst.* **12**, e2020MS002154 (2020).

77. D. M. Lawrence, R. A. Fisher, C. D. Koven, K. W. Oleson, S. C. Swenson, G. Bonan, N. Collier, B. Ghimire, L. van Kampenhout, D. Kennedy, E. Kluzek, P. J. Lawrence, F. Li, H. Li, D. Lombardozzi, W. J. Riley, W. J. Sacks, M. Shi, M. Vertenstein, W. R. Wieder, C. Xu, A. A. Ali, A. M. Badger, G. Bisht, M. van den Broeke, M. A. Brunke, S. P. Burns, J. Buzan, M. Clark, A. Craig, K. Dahlin, B. Drewniak, J. B. Fisher, M. Flanner, A. M. Fox, P. Gentine, F. Hoffman, G. Keppel-Aleks, R. Knox, S. Kumar, J. Lenaerts, L. R. Leung, W. H. Lipscomb, Y. Lu, A. Pandey, J. D. Pelletier, J. Perket, J. T. Randerson, D. M. Ricciuto, B. M. Sanderson, A. Slater, Z. M. Subin, J. Tang, R. Q. Thomas, M. Val Martin, X. Zeng, The Community Land Model version 5: Description of new features, benchmarking, and impact of forcing uncertainty. *J. Adv. Model. Earth Syst.* **11**, 4245–4287 (2019).

78. K. Riahi, D. P. van Vuuren, E. Kriegler, J. Edmonds, B. C. O'Neill, S. Fujimori, N. Bauer, K. Calvin, R. Dellink, O. Fricko, W. Lutz, A. Popp, J. C. Cuaresma, S. Kc, M. Leimbach, L. Jiang, T. Kram, S. Rao, J. Emmerling, K. Ebi, T. Hasegawa, P. Havlik, F. Humpenöder, L. A. Da Silva, S. Smith, E. Stehfest, V. Bosetti, J. Eom, D. Gernaat, T. Masui, J. Rogelj, J. Strefler, L. Drouet, V. Krey, G. Luderer, M. Harmsen, K. Takahashi, L. Baumstark, J. C. Doelman, M. Kainuma, Z. Klimont, G. Marangoni, H. Lotze-Campen, M. Obersteiner, A. Tabeau, M. Tavoni, The Shared Socioeconomic Pathways and their energy, land use, and greenhouse gas emissions implications: An overview. *Glob. Environ. Chang.* **42**, 153–168 (2017).

79. P. Landschützer, G. G. Laruelle, A. Roobaert, P. Regnier, A uniform pCO_2 climatology combining open and coastal oceans. *Earth Syst. Sci. Data* **12**, 2537–2553 (2020).

80. L. Gregor, N. Gruber, OceanSODA-ETHZ: A global gridded data set of the surface ocean carbonate system for seasonal to decadal studies of ocean acidification. *Earth Syst. Sci. Data* **13**, 777–808 (2021).

81. T. T. T. Chau, M. Gehlen, F. Chevallier, A seamless ensemble-based reconstruction of surface ocean pCO_2 and air–sea CO_2 fluxes over the global coastal and open oceans. *Biogeosciences* **19**, 1087–1109 (2022).

82. R. Wanninkhof, Relationship between wind speed and gas exchange over the ocean revisited. *Limnol. Oceanogr. Methods* **12**, 351–362 (2014).

83. S. C. Doney, I. Lima, R. A. Feely, D. M. Glover, K. Lindsay, N. Mahowald, J. K. Moore, R. Wanninkhof, Mechanisms governing interannual variability in upper-ocean inorganic carbon system and air–sea CO_2 fluxes: Physical climate and atmospheric dust. *Deep-Sea Res. II Top. Stud. Oceanogr.* **56**, 640–655 (2009).

84. N. S. Lovenduski, N. Gruber, S. C. Doney, I. D. Lima, Enhanced CO_2 outgassing in the Southern Ocean from a positive phase of the Southern Annular Mode. *Global Biogeochem. Cycles* **21**, 2900 (2007).

85. A. Mignot, K. von Schuckmann, P. Landschützer, F. Gasparin, S. van Gennip, C. Perruche, J. Lamouroux, T. Amm, Decrease in air-sea CO_2 fluxes caused by persistent marine heatwaves. *Nat. Commun.* **13**, 4300 (2022).

86. J. L. Sarmiento, N. Gruber, *Ocean Biogeochemical Dynamics* (Princeton University Press, 2006).

87. N. Goris, J. Tjiputra, J. Schwinger, C. Heinze, Responses of carbon uptake and oceanic pCO_2 to climate change in the North Atlantic: A model study with the Bergen Earth System Model. *Global Biogeochem. Cycles* **29**, 1567–1583 (2015).

88. M. P. Humphreys, E. R. Lewis, J. D. Sharp, D. Pierrot, PyCO2SYS v1. 8: Marine carbonate system calculations in Python. *Geosci. Model Dev.* **15**, 15–43 (2022).

89. T. Ziehn, M. A. Chamberlain, R. M. Law, A. Lenton, R. W. Bodman, M. Dix, L. Stevens, Y.-P. Wang, J. Srbinovsky, The Australian Earth System Model: ACCESS-ESM1.5. *J. South. Hemisph. Earth Syst. Sci.* **70**, 193–214 (2020).

90. N. C. Swart, J. N. S. Cole, V. V. Kharin, M. Lazare, J. F. Scinocca, N. P. Gillett, J. Anstey, V. Arora, J. R. Christian, S. Hanna, Y. Jiao, W. G. Lee, F. Majaess, O. A. Saenko, C. Seiler, C. Seinen, A. Shao, M. Sigmond, L. Solheim, K. von Salzen, D. Yang, B. Winter, The Canadian Earth System Model version 5 (CanESM5.0.3). *Geosci. Model Dev.* **12**, 4823–4873 (2019).

91. O. Gutjahr, D. Putrasahan, K. Lohmann, J. H. Jungclaus, J.-S. von Storch, N. Brüggemann, H. Haak, A. Stössel, Max Planck Institute Earth System Model (MPI-ESM1.2) for the High-Resolution Model Intercomparison Project (HighResMIP). *Geosci. Model Dev.* **12**, 3241–3281 (2019).

92. T. Hajima, M. Watanabe, A. Yamamoto, H. Tatebe, M. A. Noguchi, M. Abe, R. Ohgaito, A. Ito, D. Yamazaki, H. Okajima, A. Ito, K. Takata, K. Ogochi, S. Watanabe, M. Kawamiya, Development of the MIROC-ES2L Earth system model and the evaluation of biogeochemical processes and feedbacks. *Geosci. Model Dev.* **13**, 2197–2244 (2020).

93.. Seland, M. Bentsen, D. Olivié, T. Toniazzo, A. Gjermundsen, L. S. Graff, J. B. Debernard, A. K. Gupta, Y.-C. He, A. Kirkevåg, J. Schwinger, J. Tjiputra, K. S. Aas, I. Bethke, Y. Fan, J. Griesfeller, A. Grini, C. Guo, M. Ilicak, I. H. H. Karset, O. Landgren, J. Liakka, K. O. Moseid, A. Nummelin, C. Spensberger, H. Tang, Z. Zhang, C. Heinze, T. Iversen, M. Schulz, Overview of the Norwegian Earth System Model (NorESM2) and key climate response of CMIP6 DECK, historical, and scenario simulations. *Geosci. Model Dev.* **13**, 6165–6200 (2020).

94. J. F. Tjiputra, J. Schwinger, M. Bentsen, A. L. Morée, S. Gao, I. Bethke, C. Heinze, N. Goris, A. Gupta, Y.-C. He, D. Olivié, Ø. Seland, M. Schulz, Ocean biogeochemistry in the Norwegian Earth System Model version 2 (NorESM2). *Geosci. Model Dev.* **13**, 2393–2431 (2020).

95. M. Kelley, G. A. Schmidt, L. S. Nazarenko, S. E. Bauer, R. Ruedy, G. L. Russell, A. S. Ackerman, I. Aleinov, M. Bauer, R. Bleck, V. Canuto, G. Cesana, Y. Cheng, T. L. Clune, B. I. Cook, C. A. Cruz, A. D. Del Genio, G. S. Elsaesser, G. Faluvegi, N. Y. Kiang, D. Kim, A. A. Lacis, A. Leboissetier, A. N. LeGrande, K. K. Lo, J. Marshall, E. E. Matthews, S. McDermid, K. Mezuman, R. L. Miller, L. T. Murray, V. Oinas, C. Orbe, C. P. García-Pando, J. P. Perlwitz, M. J. Puma, D. Rind, A. Romanou, D. T. Shindell, S. Sun, N. Tausnev, K. Tsigaridis, G. Tselioudis, E. Weng, J. Wu, M.-S. Yao, GISS-E2.1: Configurations and climatology. *J. Adv. Model. Earth Syst.* **12**, e2019MS002025 (2020).

96. A. A. Sellar, C. G. Jones, J. P. Mulcahy, Y. Tang, A. Yool, A. Wiltshire, F. M. O'Connor, M. Stringer, R. Hill, J. Palmieri, S. Woodward, L. de Mora, T. Kuhlbrodt, S. T. Rumbold, D. I. Kelley, R. Ellis, C. E. Johnson, J. Walton, N. L. Abraham, M. B. Andrews, T. Andrews, A. T. Archibald, S. Berthou, E. Burke, E. Blockley, K. Carslaw, M. Dalvi, J. Edwards, G. A. Folberth, N. Gedney, P. T. Griffiths, A. B. Harper, M. A. Hendry, A. J. Hewitt, B. Johnson, A. Jones, C. D. Jones, J. Keeble, S. Liddicoat, O. Morgenstern, R. J. Parker, V. Predoi, E. Robertson, A. Siahaan, R. S. Smith, R. Swaminathan, M. T. Woodhouse, G. Zeng, M. Zerroukat, UKESM1: Description and Evaluation of the U.K. Earth System Model. *J. Adv. Model. Earth Syst.* **11**, 4513–4558 (2019).
